# Emotion recognition based on microstate analysis from temporal and spatial patterns of electroencephalogram

**DOI:** 10.3389/fnins.2024.1355512

**Published:** 2024-03-14

**Authors:** Zhen Wei, Hongwei Li, Lin Ma, Haifeng Li

**Affiliations:** School of Computer Science and Technology, Faculty of Computing, Harbin Institute of Technology, Harbin, China

**Keywords:** electroencephalogram, microstate analysis, affective computing, emotion recognition, evoked emotions

## Abstract

**Introduction:**

Recently, the microstate analysis method has been widely used to investigate the temporal and spatial dynamics of electroencephalogram (EEG) signals. However, most studies have focused on EEG at resting state, and few use microstate analysis to study emotional EEG. This paper aims to investigate the temporal and spatial patterns of EEG in emotional states, and the specific neurophysiological significance of microstates during the emotion cognitive process, and further explore the feasibility and effectiveness of applying the microstate analysis to emotion recognition.

**Methods:**

We proposed a KLGEV-criterion-based microstate analysis method, which can automatically and adaptively identify the optimal number of microstates in emotional EEG. The extracted temporal and spatial microstate features then served as novel feature sets to improve the performance of EEG emotion recognition. We evaluated the proposed method on two publicly available emotional EEG datasets: the SJTU Emotion EEG Dataset (SEED) and the Database for Emotion Analysis using Physiological Signals (DEAP).

**Results:**

For the SEED dataset, 10 microstates were identified using the proposed method. These temporal and spatial features were fed into AutoGluon, an open-source automatic machine learning model, yielding an average three-class accuracy of 70.38% (±8.03%) in subject-dependent emotion recognition. For the DEAP dataset, the method identified 9 microstates. The average accuracy in the arousal dimension was 74.33% (±5.17%) and 75.49% (±5.70%) in the valence dimension, which were competitive performance compared to some previous machine-learning-based studies. Based on these results, we further discussed the neurophysiological relationship between specific microstates and emotions, which broaden our knowledge of the interpretability of EEG microstates. In particular, we found that arousal ratings were positively correlated with the activity of microstate C (anterior regions of default mode network) and negatively correlated with the activity of microstate D (dorsal attention network), while valence ratings were positively correlated with the activity of microstate B (visual network) and negatively correlated with the activity of microstate D (dorsal attention network).

**Discussion:**

In summary, the findings in this paper indicate that the proposed KLGEV-criterion-based method can be employed to research emotional EEG signals effectively, and the microstate features are promising feature sets for EEG-based emotion recognition.

## 1 Introduction

In recent years, affective computing has become an emerging direction in the field of brain-inspired intelligence. Researchers aim to enable intelligent systems to recognize, perceive, infer and interpret human emotions (Poria et al., 2017), and aspire to develop “emotional machines” with human-like emotions. Emotion is a complex psychological state. Psychologists proposed several typical theories to model human emotion: the basic emotion model, the dimensional emotion model and the constructed emotion theory. Ekman believed that human beings have six fundamental discrete emotions: sadness, joy, fear, anger, surprise, and disgust (Ekman and Friesen, 1971). The most widely used dimensional model is the circumplex model of affect proposed by Russell and Barrett (1999), which uses only valence and arousal dimensions to model emotions. The theory of constructed emotion proposed by Barrett (2017) proposes that emotions should be modeled holistically, as whole brain–body phenomena in context. The theory views emotions as constructions of the world, rather than reactions to it. In the field of cognitive neuroscience, event-related potential (ERP) components with short (N100 and P100) to medium (N200 and P200) latency are demonstrated to be correlated with valence, whereas medium to long latency components (P300 and late positive potential) are shown to correlate with arousal (Hajcak et al., 2010; Kim et al., 2013). Neuroimaging studies with positron emission tomography (PET) and functional magnetic resonance imaging (fMRI) have shown that [As reviewed in Phan et al. (2002)]: the medial prefrontal cortex, the anterior cingulate, the amygdala and the insula are essential brain areas in emotional information processing; sadness was associated with activity in the subcallosal cingulate and the occipital cortex and the amygdala are activated by visual emotional stimuli.

Emotion recognition is one of the core topics in the field of affective computing, aiming to detect the emotional state of human beings from subjective experiences, neurophysiological signals, and external emotional expressions (Alarcao and Fonseca, 2019). Among the commonly used neurophysiological signals, Electroencephalography (EEG) has been widely used in the fields of emotion recognition due to its excellent time resolution (millisecond level) and non-invasiveness. There are usually two strategies for EEG emotion recognition: step-by-step machine learning and end-to-end deep learning (Zhang et al., 2020). The step-by-step machine learning strategy mainly involves three steps: EEG data acquisition and preprocessing, feature extraction and machine-learning-based classification. Generally, features from EEG can be divided into time domain, frequency domain, time-frequency domain and spatial domain. The time domain features can capture the dynamic characteristics and temporal variation trends of unstable EEG signals, such as statistical features and entropy features (Nawaz et al., 2020). The frequency domain features describe the periodicity characteristic of EEG signals, including differential entropy (Zheng et al., 2019), power spectral density (Li X. et al., 2019) and so on. The commonly used feature extraction methods in time-frequency domain include wavelet transform (Subasi et al., 2021), empirical mode decomposition (EMD) (Mert and Akan, 2018) and so on, which combine the temporal and spatial information of EEG. Besides, common spatial pattern (CSP) (Hu et al., 2022) and hierarchical discriminant component analysis (HDCA) are popular feature extraction methods which focus on relationship between electrodes and specific brain regions. In order to describe emotion in a more comprehensive way from different perspectives, researchers usually combine various feature extraction strategies to improve the performance of emotion recognition (Li et al., 2018). With the wide application of deep learning strategies, the accuracy of EEG-based emotion recognition is getting increasingly higher (Zhang et al., 2020) investigated the application of several deep learning models to the EEG-based emotion recognition, including deep neural networks (DNN), convolutional neural networks (CNN), long short-term memory (LSTM), and a hybrid model of CNN and LSTM (CNN-LSTM). The results showed that the hybrid CNN-LSTM model achieved the highest accuracy of 94.17% on the raw DEAP dataset. Recently, graph neural networks (GNN) have shown excellent performance in EEG emotion recognition (Zhang et al., 2022; Pan et al., 2024), which regard EEG signals as graph-structured data and extract high-level spatiotemporal information from EEG. Besides, some deep learning training strategies, such as domain adaptation (He et al., 2022) and transfer learning (Li J. et al., 2019), are highly favored especially in cross-subject EEG emotion recognition.

These previous studies using time and frequency domain features have achieved great success in EEG-based emotion recognition. However, these features mainly reflect the characteristics of localized brain activities, failing to describe the global working mode of the brain during the affective process. In addition, EEG is a non-stationary and fast-changing voltage signal, which results in dramatic and rapid changes in features extracted from EEG, whereas emotion states change gradually and gently (Chen et al., 2021). Most existing feature extraction methods ignore these differences between emotion and EEG signals. On account of these aspects, we propose a feature extraction method based on EEG microstates for emotion recognition, which can capture the temporal and spatial dynamics of EEG from a global perspective.

The microstate analysis technique is based on scalp topographic maps clustering, which had been proven to be effective to capture the rich spatial–temporal information in EEG signals, and can reflect the global functional network activity of the brain (Khanna et al., 2015; Michel and Koenig, 2018; Tarailis et al., 2023). Lehmann et al. (1987) showed that the time series of scalp potential topographic maps of spontaneous EEG signal do not change continuously or randomly over time, but remain stable within a certain period typically ranging from 80 to 120 milliseconds, followed by an abrupt alteration into a new configuration which returns its stability (Michel and Koenig, 2018). The scalp electric potential can reflect the instantaneous state of global activity of the underlying brain functional network, and the changes in topographical configuration indicate the transformation of the global cooperation mode of the brain functional network. The stages at which these topographic maps remain in a stable state are called “functional microstates” (Pascualmarqui et al., 1995; Lehmann et al., 1998; Khanna et al., 2015), which reflect the basic steps of information processing in the human brain.

The key challenge of utilizing microstate analysis to study EEG signals in emotional states is how to determine the optimal number of microstates. In resting-state EEG signals, despite the different clustering algorithms and datasets, researchers commonly identify four clusters (i.e., microstates). These four microstate categories exhibit highly similar configurations across studies (Michel and Koenig, 2018; Tarailis et al., 2023). Thus, many studies tend to fix the number of microstates at four to keep consistent with previous studies.

However, since the EEG signal in the emotional state contains the dynamics of emotion and other emotion-related cognitive processes, it is more complex compared with the EEG in the resting state. It is necessary to combine various optimization criteria to determine the optimal number of microstates quantitatively for the emotional EEG. Commonly used optimization criteria in resting EEG include global explained variance (GEV), cross-validation (CV) criterion, dispersion criterion, Krzanowski-Lai (KL) criterion, and the normalized KL criterion (Murray et al., 2008; Michel and Koenig, 2018; Poulsen et al., 2018). GEV is considered to represent the proportion of data that can be interpreted by all microstate classes, which is used to evaluate the quality of clustering. However, compromise between clustering quality and data reduction is needed when using the GEV criterion. Dispersion is a measure of intra-cluster similarity, but it cannot be used in the clustering methods which are polarity-invariant, such as modified K-means. Both the KL criterion (Krzanowski and Lai, 1988) and the normalized KL criterion are essentially a method to find the “elbow” of the dispersion curve. The “elbow” refers to the point of highest deceleration where adding additional one more microstate will not increase the quality of the results (Murray et al., 2008; Poulsen et al., 2018). Inspired by the GEV and KL criteria, we proposed a KL_GEV_ criterion here, to address the core problem of determining the optimal number of microstates in emotional EEG. The core idea of the KL_GEV_ criterion is to find the “elbow point” (L-corner) of the GEV curve, in other words, the inflection point between the rapid growing period and the flat period of the GEV curve.

**Figure 1:**
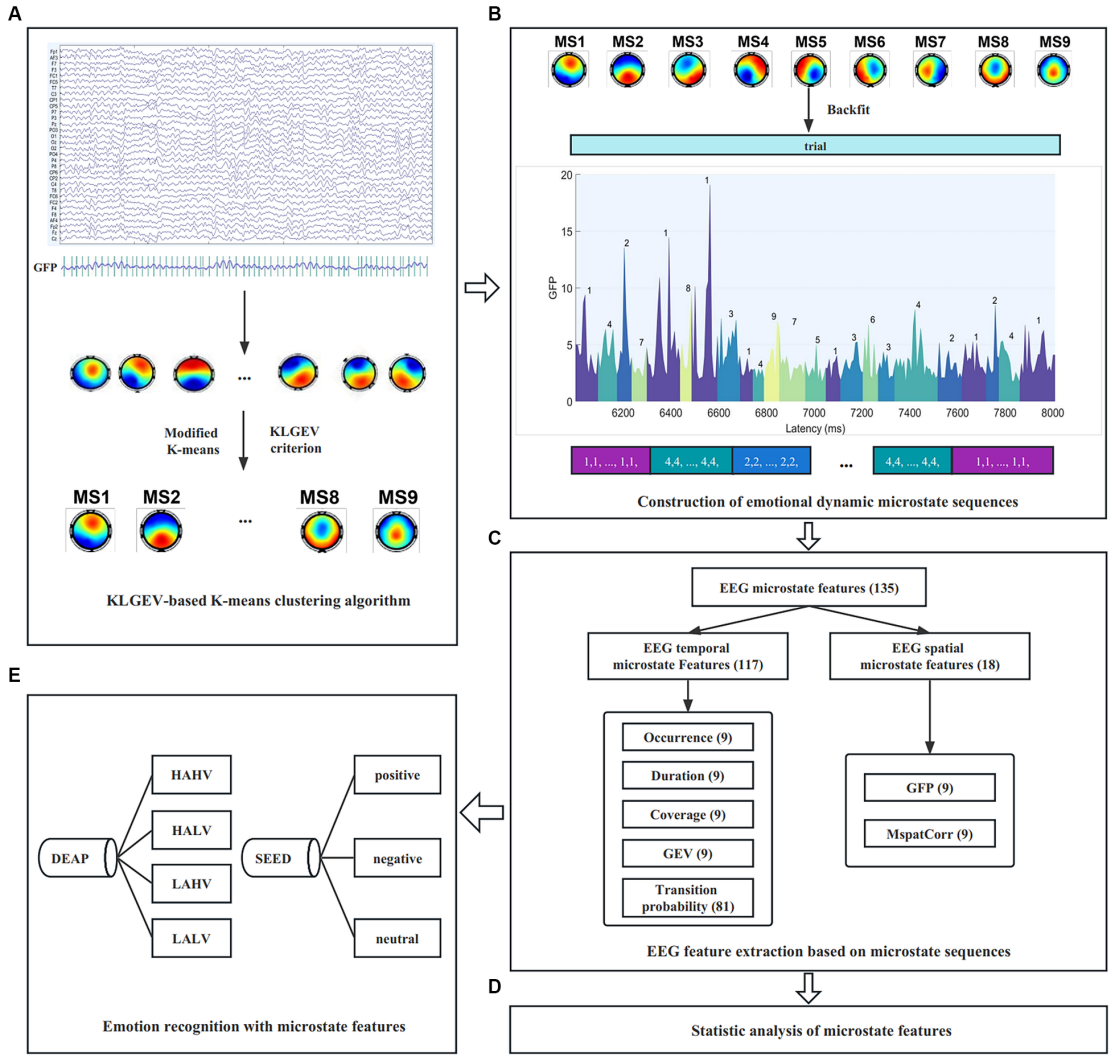
The schema of the study. **(A)** Spatial clustering of topographic maps across subjects using the proposed KL_GEV_-based K-means clustering algorithm. **(B)** Construction of emotional dynamic microstate sequences. **(C)** Microstate temporal and spatial feature extraction from the microstate sequences (Here we take the DEAP dataset as an example). **(D)** Statistical analysis of microstate features to characterize spatiotemporal dynamics under different emotional states. **(E)** Emotion recognition with microstate features on the SEED and DEAP datasets.

The work in this paper mainly includes the following three aspects: (1) We proposed a KL_GEV_-criterion-based microstate analysis method based on the GEV and KL criteria, which can automatically and adaptively determine the optimal number of microstates in emotional EEG signals, so as to explore the global working mode of the brain during the occurrence and evolution of emotion. Sufficient experiments were carried out on two public emotional datasets (2) We introduced two microstate spatial parameters (Poulsen et al., 2018) on the basis of the five commonly used temporal parameters. These parameters were used as feature sets for emotion recognition on two benchmark datasets SEED and DEAP, yielding good performance (3) We performed statistical analysis on the seven microstate parameters, to investigate the spatiotemporal dynamic characteristics of EEG signals under different emotional states. The results partially revealed the specific neurophysiological significance of microstates during the emotional cognitive process, and broaden our knowledge of the functional interpretability of microstates. The schema of the present study is shown in Figure 1.

## 2 Materials and methods

### 2.1 Electroencephalogram datasets and preprocessing

Two public emotional EEG datasets were used for microstate analysis: the SJTU Emotion EEG Dataset (SEED) (Duan et al., 2013; Zheng and Lu, 2015) and Database for Emotion Analysis using Physiological Signals (DEAP) (Koelstra et al., 2012).

#### 2.1.1 Dataset 1: the SJTU emotion electroencephalogram dataset

The SEED dataset contains EEG data of 15 subjects when they were watching different types (positive, negative, and neutral emotions) of film clips. The EEG was continuously recorded with 62-channel ESI NeuroScan System at a sampling rate of 1,000 Hz. Each subject performed the experiment three times with an interval of about one week, for a total of 45 sessions. Each session consists of 15 trials, in which subjects were asked to watch a film clip lasting about four minutes. We carefully scrolled and reviewed the EEG data from 45 sessions, and removed 5 of them with lots of noise and artifacts. As a result, 40 sessions in SEED were used for subsequent processing and analysis.

A standard preprocessing pipeline was conducted for artifact removal. Firstly, we applied a bandpass filter of 1–45 Hz for the desired frequency range and a notch filter of 48–52 Hz for power line noise removal to each session of EEG data. Secondly, the filtered EEG data were common average referenced. Thirdly, the EEG was down-sampled to 200 Hz. Finally, we removed the artifacts from the eyes and muscles using independent component analysis (ICA).

#### 2.1.2 Dataset 2: database for emotion analysis using physiological signals

The DEAP dataset contains EEG data of 32 subjects when they were watching music video clips. The EEG was collected with 32-channel Biosemi ActiveTwo system at a sampling rate of 512 Hz. Each experiment consists of 15 trials, in which subjects were asked to watch a one-minute music video clip and fill out a self-assessment mood scale after watching. Each video is scored on the dimensions of arousal and valence, which are rated on a continuous scale ranging from 1 to 9.

Since the EEG data from subjects No. 1–22 and No. 23–32 in the DEAP dataset were collected under different hardware conditions, only No. 1–22 were selected in this study to exclude the influence of different experimental conditions. In addition, the EEG data of No. 1–22 were further scrolled and examined, and two (subjects 8 and 17) with lots of noise and artifacts were removed. As a result, we used the EEG data from 20 subjects for further processing and analysis. The preprocessing procedure of the DEAP is the same as that of the SEED, with a replaced down-sampling step to 256 Hz after common average referencing.

### 2.2 The proposed KL_GEV_-criterion-based microstate analysis

Based on the modified K-means spatial clustering algorithm, we proposed a KL_GEV_-criterion-based microstate analysis method, which can automatically and adaptively determine the optimal number of microstates in emotional EEG signals. The proposed method was used to construct the microstate time series, so as to capture important spatiotemporal dynamics of EEG signals during the affective process.

#### 2.2.1 Global field power

EEG microstates are defined as successive short time periods (or stages) during which the configuration of the scalp potential field remains semi-stable (Michel and Koenig, 2018). Before clustering of the original topographic maps, the global field power (GFP) at each time point in the EEG signal is calculated. The scalp potential maps at the peak point of GFP curves are used as the original maps of the spatial clustering algorithm. GFP is calculated as follows:


(1) $$GFP_{n}=\sqrt{\frac{\sum_{i=1}^{C}{\left[v_{i}\left(n\right)-\bar{v}\left(n\right)\right]}^{2}}{C}}$$

where *C* represents the number of electrodes, $v_{i}\left(n\right)$ is the measured voltage of a specific electrode *i* at sampling point *n*, and $\bar{v}\left(n\right)$ is the average voltage of all *C* electrodes at the respective sampling point *n*.

Mathematically, GFP equals the root mean square across the average-referenced electrode values at a given instant in time, i.e., the standard deviation of all electrodes at a given time. GFP provides a single and reference-independent measure of response strength of topographic maps (Lehmann and Skrandies, 1980). The local maxima of the GFP curve are considered to have stable topological configuration and high signal-to-noise ratio, whereas topographic maps with low GFP tend to have low signal-to-noise ratio, which means the topographical configuration is changing from one to another (Murray et al., 2008). As a result, only the topographic maps at the GFP peak point are selected as the original maps for the spatial clustering algorithm.

#### 2.2.2 KL_GEV_-based K-means clustering algorithm

**Figure 2:**
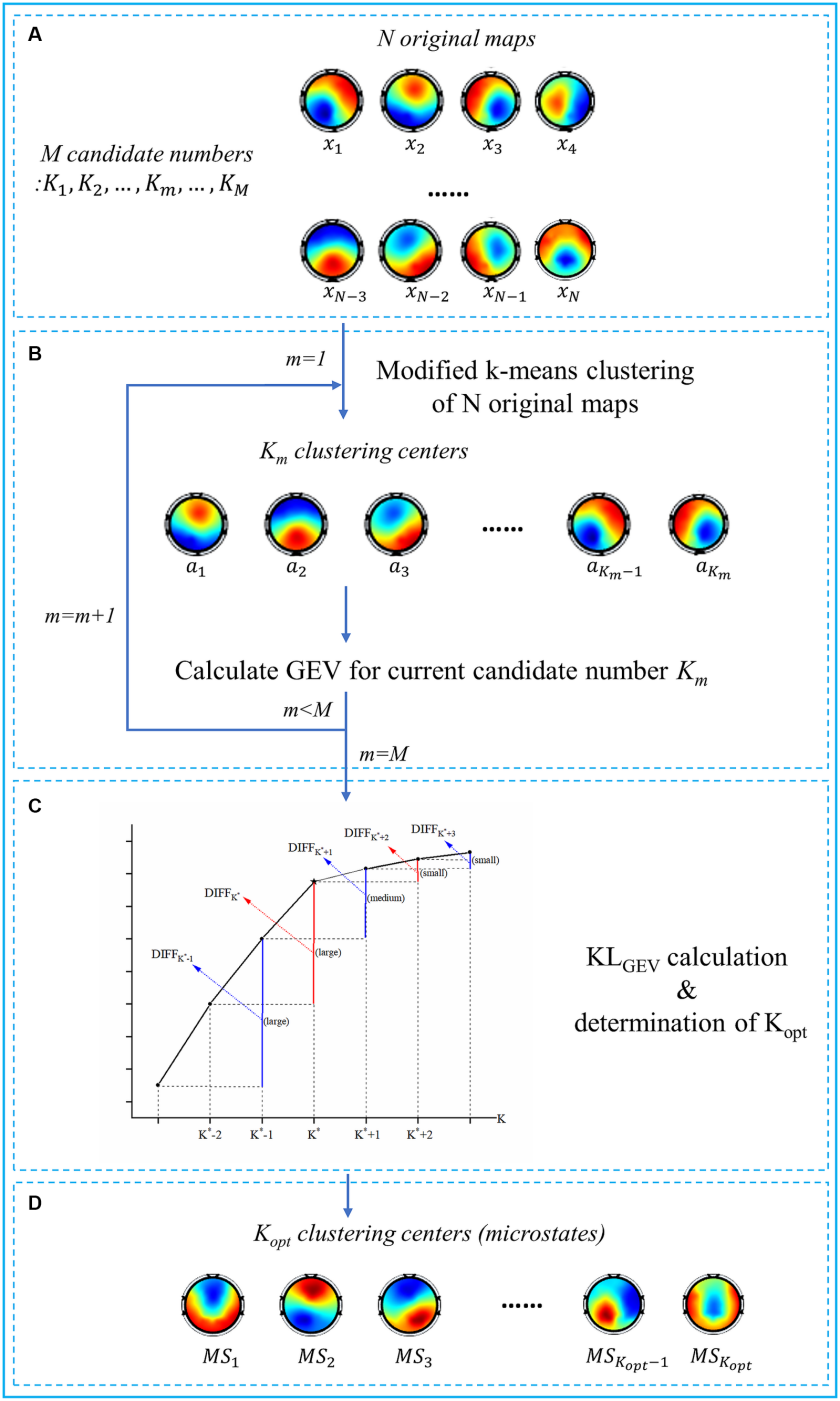
Flowchart of the proposed KL_GEV_-criterion-based algorithm. **(A)** The inputs of the algorithm include N original topographic maps and M candidate numbers. The original maps are defined as the scalp potential maps at the peak point of GFP curves. **(B)** For each candidate number $K_{m}$ , the modified K-means clustering algorithm is used. The N original topographic maps are thus clustered into $K_{m}$ clustering centers. We then calculate GEV for each candidate number as a preparation for the KL_GEV_ criterion. **(C)** The schematic diagram of the KL_GEV_ criterion and determination of the $K_{\mathrm{optimal}}$ . K^*^ is regarded as the ‘elbow point’ (the star) of the GEV curve, i.e., the local peak points of the KL_GEV_ curve. According to the KL_GEV_ criterion, the largest local peak point of the KL_GEV_ curve is determined as the optimal one. **(D)** The outputs of the algorithm are the identified $K_{\mathrm{optimal}}$ clustering centers, i.e., the optimal microstate classes.

Based on the modified K-means spatial clustering algorithm, we proposed a KL_GEV_-criterion-based microstate analysis method in this paper to automatically and adaptively determine the optimal number of microstates in emotional EEG signals. The flowchart of the proposed algorithm is shown in Figure 2, to provide a clear and concise depiction of the steps involved in the algorithm.

Global explained variance (GEV) is considered to represent the proportion of data that can be interpreted by all microstate classes. GEV is commonly used to evaluate the quality of clustering. Theoretically, a higher GEV stands for a better clustering result, which means that the current K kinds of microstates can explain a higher proportion of the data. The GEV of the current K clusters is calculated by Equation (2), which is equal to the sum of the global explained variance $GEV_{k}$ of all clusters. The global explained variance of each cluster is calculated by Equation (3), which equals to the sum of the global explained variance of all sampling points with cluster label *k*:


(2) $$GEV=\overset{K}{\underset{k}{\sum }}GEV_{k}$$

(3) $$GEV_{k}=\overset{N_{k}}{\underset{n}{\sum }}GEV_{n},for\;l_{n}=k$$

where $N_{k}$ refers to the number of sampling points assigned to cluster *k*, and $l_{n}$ is the microstate label of the potential topographic map at sampling point *n*.

The global explained variance at each sampling point is calculated by Equation (4), which reflects the spatial similarity between the potential topographic map $x_{n}$ at each sampling point and the microstate template map (cluster center) $a_{l_{n}}$ to which $x_{n}$ belongs:


(4) $$GEV_{n}=Corr_{x_{n},a_{l_{n}}}^{2}\frac{GFP_{n}^{2}}{\sum_{n^{\prime }=1}^{N}GFP_{n^{\prime }}^{2}}$$

where $Corr_{x_{n},{a_{l}}_{n}}$ is the spatial correlation coefficient between $x_{n}$ and $a_{l_{n}}$ . $GFP_{n}$ and $GFP_{n^{\prime }}$ represent the global field power at sampling points *n* and $n^{\prime }$ respectively, calculated by Equation (1). N is the number of all sampling points.

As GEV will increase with the number of microstates (i.e., the number of clusters), a larger GEV usually corresponds to a larger number of microstates. Excessive microstates can result in high similarity between each microstate and fail to reflect the activity characteristics of different neuronal assemblies. In order to make a compromise between clustering quality and data reduction, the KL criterion was introduced in this paper to find the “elbow point” (L-corner) of the GEV curve, to automatically determine the optimal number of microstates (i.e., the optimal number of clusters) in emotional EEG. The “elbow” is the point where the growth of GEV is significantly reduced, in other words, where the increase in GEV caused by adding one more microstate decreases significantly.

To find the optimal number of microstates, we need to find the inflection point between the rapid growing period and the flat period of the GEV curve. The KL_GEV_ criterion investigates the first-order difference of GEV curve with microstate number interval of 2. Compared with the interval of 1, it can reduce the influence of irregular local jitter on the curve, and reflect more clearly and accurately the overall trend of the GEV curve. Let $DIFF_{K}$ denotes the first-order discrete difference with interval 2 in the function $K^{\left(2/C\right)}GEV_{K}$ when the number of groups in the clustering is increased from K-2 to K, i.e.,


(5) $$DIFF_{K}=K^{\left(2/C\right)}GEV_{K}-{\left(K-2\right)}^{\left(2/C\right)}GEV_{K-2}$$

where *C* is the number of electrodes, and $GEV_{K}$ refers to the global explained variance when the candidate number of microstates is *K*, calculated by Equation (2).

Then we would expect GEV to increase dramatically as K is increased, as long as K is less than the optimal number K*, but this increase should slow down after K = K*. Thus, we would expect that (as shown in Figure 2C):

(i) For K < K^*^, both $DIFF_{K}$ and $DIFF_{K+2}$ should be large (or medium) and positive;
(ii) For K > K^*^, both $DIFF_{K}$ and $DIFF_{K+2}$ should be small (or medium) and positive;
(iii) For K = K^*^, $DIFF_{K^{*}}$ should be large and positive, while $DIFF_{K^{*}+2}$ should be relatively small and positive.

On the basis of the above expectation, therefore, a reasonable criterion to determine the optimal number of microstates automatically is:


(6) $$KL_{GEV}=\frac{DIFF_{K}}{DIFF_{K+2}}$$

As a consequence, the local peak points of the KL_GEV_ curve correspond to the elbow of the GEV curve. In practice, there are usually several local peak points on the KL_GEV_ curve, and the KL_GEV_ criterion identify the largest local peak point as the one indicating the optimal number of microstates.

The clustering algorithm includes two steps: reassigning and recalculation. During the reassigning step, the algorithm determines the category $l_{n}$ for each original topographic map $x_{n}$ . In this step, the algorithm assigns each original topographic map to one of the K clusters. $l_{n}$ is determined using Equations (7) and (8) as follows:


(7) $$l_{n}=\underset{k}{\mathrm{argmin}}\left\{d_{kn}^{2}\right\}$$

(8) $$d_{kn}^{2}=x_{n}^{T}\cdot x_{n}-{\left(x_{n}^{T}\cdot a_{k}\right)}^{2}-\lambda b_{kn}$$

where $x_{n}$ refers to the potential vector of the original map n , $a_{k}$ is the potential vector of the kth cluster center, and $d_{kn}^{2}$ is the orthogonal square Euclidean distance between $x_{n}$ and $a_{k}$ .

The recalculation step recalculates the cluster center of each cluster, which is defined as the mathematical average of all original maps in each cluster. After the clustering algorithm is finished, all the original topographic maps are clustered into K classes, and K clustering centers (i.e., microstate template maps) are obtained.

The complete procedure of the KL_GEV_-based K-means clustering algorithm is shown in Algorithm 1. The algorithm consists of two stages: the first stage is the modified K-means spatial clustering algorithm, which obtains several candidate numbers of microstates by clustering the original topographic maps; The second stage is the identification of the optimal number of microstates from candidate numbers based on the KL_GEV_ criterion. The algorithm outputs the final cluster centers, i.e., microstate template maps.

**ALGORITHM 1:** KL_GEV_-based K-means clustering algorithm.

***Input:***	*Set of N original topographic maps:* $D=\left\{x_{1},x_{2},\ldots ,x_{n},\ldots ,x_{N}\right\}$ , *Set of M candidate numbers of microstates:* $K:\mathrm{range}=\left\{K_{1},K_{2},\ldots ,K_{m},\ldots ,K_{M}\right\}$ , *Maximum number of iterations:* max:Ite *.*
***Output:***	*The optimal number of microstates:* $K_{\mathrm{optimal}}$ , *Cluster centers (*i.e.*, microstate template maps):* $\mathrm{ClusterCerters}=\left\{a_{1},a_{2},\ldots ,a_{k},\ldots ,a_{K_{\mathrm{optimal}}}\right\}$
*1:*	$GEV:\mathrm{list}=\left[\right],\mathrm{ClusterCenters}:\mathrm{list}=\left\{\right\}$
*2:*	ForKinK:range
*3:*	***Random initialization:** K cluster centers are randomly selected from D,* $\mathrm{ClusterCenters}=\left\{a_{1},a_{2},\ldots ,a_{k},\ldots ,a_{K}\right\}$ *.*
*4:*	iteration=0
*5:*	Repeat
*6:*	iteration+=1
*7:*	***Reassign:** determine the category* $l_{n}$ *for each original topographic map* $x_{n}$ *according to* Equations (7) and (8).
*8:*	***Recalculation:** recalculate the cluster center of each cluster. The cluster center is defined as the mathematical average of all original maps in each cluster:* $\mathrm{ClusterCenters}=\left\{a_{1},a_{2},\ldots ,a_{k},\ldots ,a_{K}\right\}$
*9:*	Untiliteration≥max:Ite
*10:*	***Calculate GEV for candidate number K:** the GEV is calculated using* Equations (2, 3, 4). $GEV:\mathrm{list}\left(K\right)=GEV,\mathrm{ClusterCenters}:\mathrm{list}\left\{K\right\}=\mathrm{ClusterCenters}$ *.*
*11:*	Endfor
*12:*	***KL***_***GEV***_ ***calculation:** the KL_GEV_ of all candidate numbers of microstates are calculated using* Equations (5) and (6).
*13:*	***Determination of*** $K_{\mathrm{optimal}}$ ***:** choose the**largest local peak point** of the KL_GEV_ curve as the one indicating the optimal number of microstates.*
*14:*	$\mathrm{ClusterCenters}=\mathrm{ClusterCenters}:\mathrm{list}\left\{K_{\mathrm{optimal}}\right\}$
*15:*	***Return*** $K_{\mathrm{optimal}}$ , ClusterCerters

#### 2.2.3 Backfitting and temporal smoothing

The obtained microstate template maps are used to backfit scalp potential maps at each sampling point in EEG data based on Pearson spatial correlation coefficients. The Pearson correlation coefficient between each scalp potential map and each template map is calculated by Equation (9) as follows:


(9) $$Corr_{u,v}=\frac{\sum_{i=1}^{C}u_{i}v_{i}}{\sqrt{\sum_{i=1}^{C}u_{i}^{2}}\sqrt{\sum_{i=1}^{C}v_{i}^{2}}}$$

where *C* represents the number of electrodes, u or v refers to the potential topographic map, i.e., the potential topographic map at each sampling point or the template map, and $u_{i}$ or $v_{i}$ is the potential value of the topographic map u or v at electrode *i, respectively.*

After the calculation of spatial correlation coefficients, the topographic map at each sampling point is assigned to one template map (i.e., microstate) with the highest spatial correlation coefficient. In this way, the potential topographic maps at all sampling points in EEG signals are represented as a series of template maps, and the raw EEG signals are modeled as a time series of alternating functional microstates, which can characterize the dynamic process of the brain during affective processing.

Due to the existence of noise signals, there are usually some short-duration microstate segments in the microstate time series obtained from topographic map backfitting. We adopted the windowed smoothing algorithm proposed by Pascualmarqui et al. (1995) to smooth these small noise segments.

#### 2.2.4 Microstate temporal and spatial features

By analyzing the EEG microstate time series, several microstate parameters can be obtained (Murray et al., 2008; Michel and Koenig, 2018; Tarailis et al., 2023). We introduced two microstate spatial parameters, namely average global field power and mean spatial correlation (Poulsen et al., 2018), on the basis of the commonly used temporal parameters. The microstate temporal and spatial parameters used in this paper are summarized as follows:

(a) Occurrence: the frequency of occurrence of each microstate;
(b) Duration: the average duration (average lifespan) that a given microstate remains stable;
(c) Coverage: the time coverage rate of each microstate throughout the whole-time course, in other words, the fraction of the total recording time for which a given microstate is dominant;
(d) Transition probability between microstates classes;
(e) Global explained variance (GEV) of each microstate, which is calculated using Equation (3);
(d) Average global field power ( ${GFP}_{k}$ ) of each microstate, represented by the average global field power $GFP_{n}$ of all sampling points assigned to the *kth* microstate. ${GFP}_{k}$ is calculated by Equation (10) as follows:

(10) $$GFP_{k}=\frac{1}{N_{k}}\overset{N_{k}}{\underset{n}{\sum }}GFP_{n},for\;l_{n}=k$$

where $GFP_{n}$ is calculated using Equation (1).

(g) Mean spatial correlation (MspatCorr) of each microstate, which is the average spatial correlation between the template map of each microstate class and the potential topographic maps assigned to this microstate. It is calculated by Equation (11) as follows:


(11) $$\mathrm{MspatCor}r_{k}=\frac{1}{N_{k}}\overset{N_{k}}{\underset{n}{\sum }}Corr_{x_{n},a_{l_{n}}},for\;l_{n}=k$$

As reviewed in Murray et al. (2008), Khanna et al. (2015), Poulsen et al. (2018) and Tarailis et al. (2023), these parameters well describe the temporal and spatial dynamic characteristics of the microstate series and the EEG signals, reflecting the response strength, temporal and spatial characteristics of potential neural assemblies and nervous systems.

### 2.3 Statistical analysis of microstate features

Statistical analyses were performed to characterize the EEG microstate differences in different emotional states. Each microstate parameter was compared on the valence and arousal dimension separately. The level differences in valence describe the positive or negative degree of emotional states, whereas the arousal dimension characterizes the level of physiological activation of emotions (Russell and Barrett, 1999).

For the DEAP dataset, we first classified all emotion-evoked EEG trials into low- or high-level groups based on the self-assessment ratings of all subjects. Each trial was rated separately in the arousal and valence dimensions, where each rating was a floating-point number ranging from 1 to 9. However, the ranges of the reported self-assessment ratings could be quite different from subject to subject, due to individual-specific experience of emotions (Hu et al., 2022b). As a result, it would be unsuitable to have a fixed threshold (e.g., 5) for grouping. Therefore, this paper adopted the self-adaptive threshold reassignment method proposed by Yin et al. (2017) to determine the threshold for level grouping for each subject. The illustration of the method is shown in Supplementary Figure S1, and the obtained self-adaptive thresholds on arousal and valence dimensions for each subject are shown in Supplementary Table S1. In this way, all trials were divided into four classes: high arousal and high valence (HAHV), high arousal and low valence (HALV), low arousal and high valence (LVHA), low arousal and low valence (LALV). For the SEED dataset, each trial has an explicit emotion label: positive, negative or neutral. Trials with positive labels and negative labels were included in the statistical analysis.

Secondly, the Wilcoxon rank-sum test was used to identify whether statistically significant differences exist between high (or positive) and low (or negative) groups for each microstate class in every parameter. The significance level is set to 0.05.

### 2.4 Emotion recognition

In order to verify whether the microstate temporal and spatial parameters extracted in this paper can effectively capture the emotional characteristics of EEG signals, we employed all the parameters extracted in Section 2.3 as a feature set for the subject-dependent emotion recognition experiment. We did additional comparison experiments which utilized only temporal parameters as a feature set, so as to investigate whether the introduced spatial parameters can further improve the accuracy of emotion recognition. Besides, we also tested whether the characterization ability of the model would be further enhanced with frequency domain features. Specifically, we extracted power spectral density (PSD) features from five bands: δ (1–4 Hz), θ (4–8 Hz), α (8–12 Hz), β (12–30 Hz), and γ (30–45 Hz), and combined them with microstate temporal and spatial features for emotion recognition. The experiments were carried out on SEED and DEAP datasets.

The open-source automatic machine learning framework AutoGluon-Tabular (Erickson et al., 2020) was chosen as the classifier for emotion recognition. AutoGluon-Tabular is an easy-to-use Python library for automatic machine learning with tabular data. It automatically evaluates the performance of multiple machine learning models (e.g., KNN, random forests, XGBoost, ensemble learning models, multi-layer stack ensembling models and even self-implemented models) at the same time, and returns the classification results using the best-performing model. Unlike existing automatic machine learning frameworks that primarily focus on model/hyperparameter selection, AutoGluon-Tabular succeeds by multi-layer stack ensemble and n-repeated k-fold bagging. For each subject, a fivefold cross-validation method was adopted to obtain the final average accuracy.

## 3 Results

### 3.1 Results of KL_GEV_-criterion-based microstate analysis

We used the proposed KL_GEV_-criterion-based method to perform microstate analysis on two public emotional EEG datasets, SEED and DEAP, to evaluate the effectiveness of the proposed method.

#### 3.1.1 Determination of the K_optimal_ using the KL_GEV_ criterion

To investigate the performance of the proposed KL_GEV_ criterion, we demonstrate here how the KL_GEV_ criterion determine the optimal number of microstates K_optimal_ on the SEED and DEAP datasets. For the SEED dataset, when the candidate number of microstates is ranging from 3 to 15, the corresponding $GEV_{K}$ , $K^{\left(2/C\right)}GEV_{K}$ , $DIFF_{K}$ , and KL_GEV_ are listed in Table 1. When the number of microstates K is smaller than 10, $DIFF_{K}$ and $DIFF_{K+2}$ are relatively large (or medium); and when the K is larger than 10, $DIFF_{K}$ and $DIFF_{K+2}$ are relatively small (or medium); while when K equals to 10, $DIFF_{10}$ is relatively large and $DIFF_{12}$ is relatively small. As a consequence, the ratio of $DIFF_{10}$ to $DIFF_{12}$ tends to be larger than ratios at other points (e.g., the ratio of $DIFF_{9}$ to $DIFF_{11}$ or the ratio of $DIFF_{11}$ to $DIFF_{13}$ ). Therefore, 10 is regarded as the ‘elbow point’ of the GEV curve, i.e., the local peak point of KL_GEV_. According to the KL_GEV_ criterion, the largest local peak point 10 is chosen as the final optimal number of microstates (K_optimal_). In the same way, the K_optimal_ equals to 9 for the DEAP dataset.

**Table 1:** Determination of the K_optimal_ using the KL_GEV_ criterion on (A) SEED and (B) DEAP datasets.

K	$GEV_{K}$	$K^{\left(2/C\right)}GEV_{K}$	$DIFF_{K}$	KL_GEV_	K	$GEV_{K}$	$K^{\left(2/C\right)}GEV_{K}$	$DIFF_{K}$	KL_GEV_
(A) Determination of the K_optimal_ using the KL_GEV_ criterion on SEED dataset (62 channels).									
3	0.5403	0.5598	-	-	**10**	**0.6511**	**0.7013**	**0.0228**	**1.4497**
4	0.5723	0.5985	0.0958	1.9063	11	0.6567	0.7095	0.0191	1.2705
5	0.5968	0.6286	0.0687	1.8568	12	0.6618	0.7170	0.0157	1.1205
6	0.6123	0.6487	0.0502	1.6871	13	0.6670	0.7245	0.0150	1.2056
7	0.6251	0.6656	0.0370	1.4888	14	0.6714	0.7311	0.0140	-
8	0.6345	0.6785	0.0298	1.3059	15	0.6753	0.7370	0.0124	-
9	0.6432	0.6905	0.0249	1.3048					
(B) Determination of the K_optimal_ using the KL_GEV_ criterion on DEAP dataset (32 channels).									
3	0.5450	0.5838	-	-	10	0.6678	0.7712	0.0288	1.2891
4	0.5828	0.6355	0.1243	1.8727	11	0.6738	0.7828	0.0240	1.1883
5	0.6094	0.6738	0.0901	1.7685	12	0.6794	0.7935	0.0224	1.2214
6	0.6275	0.7019	0.0664	1.6415	13	0.6841	0.8030	0.0202	1.1754
7	0.6418	0.7248	0.0509	1.4965	14	0.6884	0.8119	0.0183	-
8	0.6519	0.7423	0.0404	1.4017	15	0.6925	0.8202	0.0172	-
**9**	**0.6614**	**0.7588**	**0.0340**	**1.4175**					

The candidate number of microstates K is ranging from 3 to 15. $GEV_{K}$ refers to the global explained variance when the candidate number of microstates is K . C is the number of electrodes, which is 62 for the SEED dataset and 32 for the DEAP. $DIFF_{K}$ denotes the first-order difference with number interval of 2 in the $K^{\left(2/C\right)}GEV_{K}$ when the number of groups in the clustering increase from K-2 to K. The KL_GEV_ is the ratio of $DIFF_{K}$ to $DIFF_{K+2}$ , and the largest local peak point of the KL_GEV_ curve is chosen as the K_optimal_ (the bold line) (Round to 4 decimal places).

#### 3.1.2 The identified optimal microstate classes

**Figure 3:**
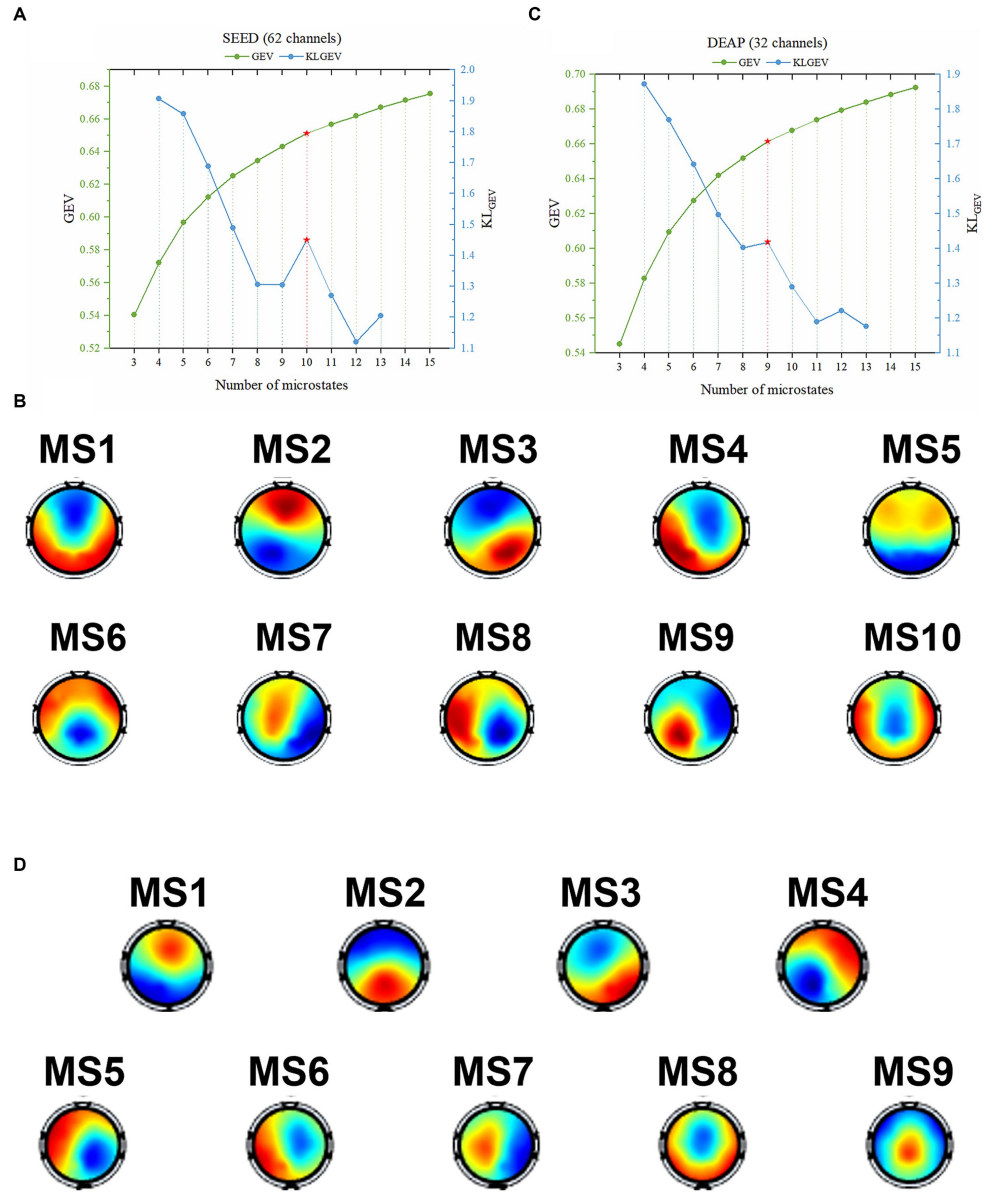
The identified optimal microstate classes using the proposed KL_GEV_ criterion for the SEED and DEAP datasets. **(A)** The GEV and corresponding KL_GEV_ values of the SEED dataset for different number of microstates. The KL_GEV_ criterion identified 10 microstates, which explained 65.11% of the data in all time points. **(B)** The final identified 10 microstate template maps from the SEED dataset. **(C)** The GEV and corresponding KL_GEV_ values of the DEAP dataset for different number of microstates. The KL_GEV_ criterion identified 9 microstates, which explained 66.14% of the data in all time points. **(D)** The final identified 9 microstate template maps from the DEAP dataset.

For the SEED dataset, the GEV curve and the corresponding KL_GEV_ obtained by the modified K-means clustering algorithm when the candidate number was from 3 to 15 were shown in Figure 3A. The corresponding template topographic maps (i.e., microstates maps) were shown in Figure 3B, which were named “MS1-MS10” respectively.

Pearson spatial correlation coefficient is a measure of the topographic similarity of microstate maps. We calculated the Pearson spatial correlation coefficient between the pairwise topographic maps within each dataset. The correlation coefficient matrix was shown in Supplementary Figure S2A. As can be seen, the similarity between each pair of the extracted microstate maps was relatively low (Most of the coefficients were less than 0.8 except for several that were slightly greater than 0.8).

Similarly, for the DEAP dataset, the GEV curve and the corresponding KL_GEV_ obtained by the modified K-means clustering algorithm were shown in Figure 3C. The corresponding template topographic maps (i.e., microstates maps) were shown in Figure 3D, which were named “MS1-MS9” respectively.

The Pearson spatial correlation coefficient matrix was shown in Supplementary Figure S2B. As can be seen, the similarity between each pair of the extracted microstate maps was relatively low (Most of the coefficients were less than 0.8 except for several that were slightly greater than 0.8).

#### 3.1.3 Corresponding relationship between microstates of two datasets

**Figure 4:**
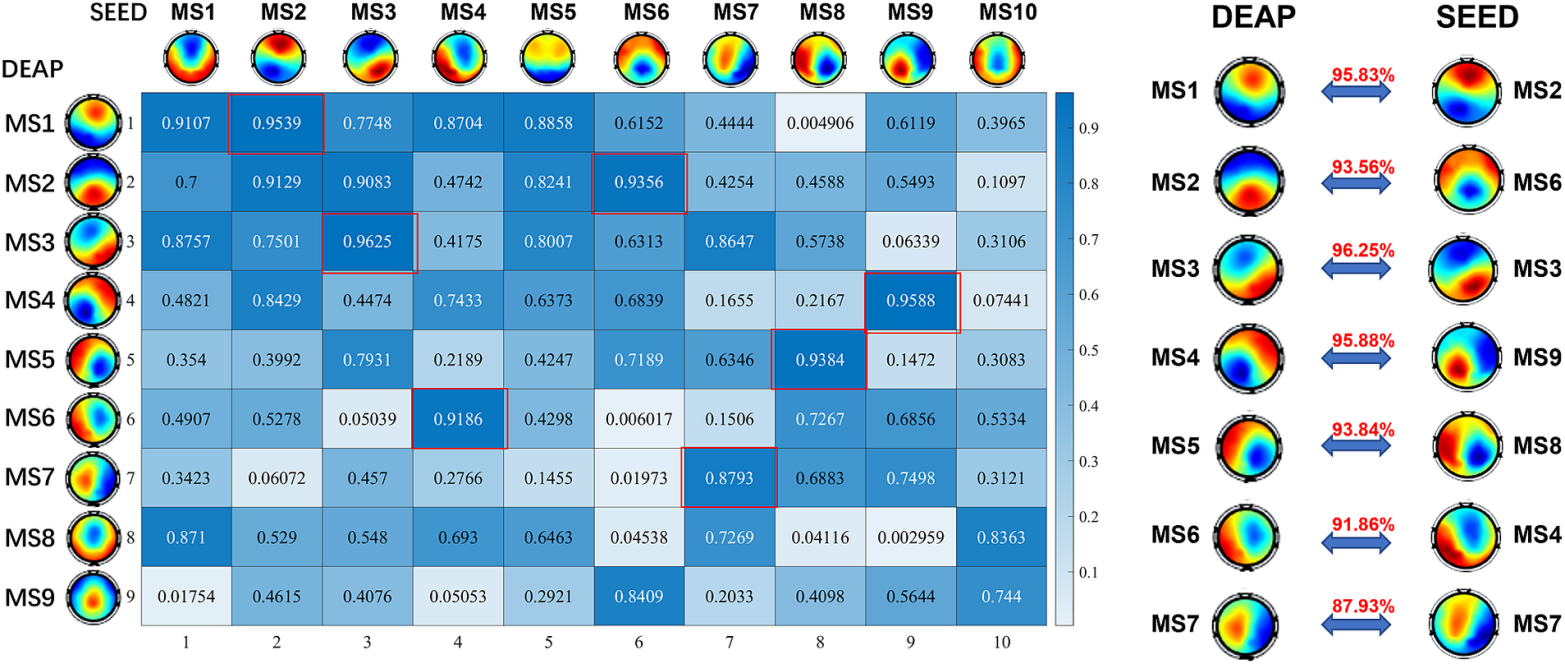
The Pearson correlation coefficients matrix between microstates of SEED and DEAP dataset. The corresponding relationship is listed on the right. These consistent microstates may represent the basic building blocks of emotion cognition.

The stimulus materials used for inducing emotion in the SEED dataset are movie clips, while the stimulus materials used in the DEAP dataset are music videos. In addition, considering the differences in subjects, number of electrodes (Zhang et al., 2021), the hardware conditions and the stability of the algorithm, the number of identified microstates in the two datasets is different. To find the correlation between the identified microstates in the two datasets, we calculated the Pearson spatial correlation coefficient between the microstate topographic maps of the two datasets, and the results are shown in Figure 4. As shown in the figure, the spatial correlation coefficient between the microstates in the SEED dataset and some of the microstates in DEAP is very high, and there is a clear correspondence. These identical functional microstates may reflect the functional patterns of the brain in the process of emotional cognition, and correspond to the basic building blocks in emotion-related information processing.

### 3.2 Spatiotemporal dynamics of EEG under different emotions

Statistical analyses were performed to characterize the EEG microstate differences, so as to investigate the spatiotemporal dynamic characteristics of EEG signals under different emotional states.

On the SEED dataset, we performed the Wilcoxon rank-sum test to identify whether statistically significant differences exist under different emotional states (positive vs. negative) for each microstate parameter. The results are shown in Supplementary Figure S3. The activities of MS2, MS3, MS4, MS8 and MS9 were significantly decreased in the positive groups compared to the negative groups, while the activities of MS1, MS5 and MS10 were significantly increased in the positive groups. Specifically, the Occurrence, Coverage and GEV of MS2 were significantly lower in the positive groups. It also showed that decreased Occurrence, Duration, Coverage and GEV of MS3 were found in the positive groups as compared with the negative groups. For MS4 and MS8, the Duration and GEV were found significantly lower in the positive groups. At last, the Occurrence and GEV of MS9 significantly decreased in the positive groups. By contrast, the Occurrence, Coverage, Duration and GEV of MS5 and MS10 were significantly increased in the positive groups compared to the negative groups. Moreover, the GEV of MS1 also significantly increased in the positive groups. In addition, all 10 microstates showed higher GFP in the positive groups, and all microstates except MS1 and MS10 had decreased MspatCorr in the positive groups as compared to the negative groups.

The transition probability between microstates can reflect the temporal dynamic characteristics of EEG signals in different emotional states, and affect the difference between microstate parameters. Supplementary Figure S3G depicted the statistically significant differences in transition probability between positive and negative groups.

In the same way, the results of the Wilcoxon rank-sum test on arousal and valence dimensions for the DEAP dataset are shown in Supplementary Figures S4, S5. For the arousal dimension, the activity of MS9 was significantly increased in the high arousal groups compared to low arousal groups, while the activity of MS7 was significantly decreased in the high groups. Specifically, the Occurrence, Coverage and GEV of MS9 were significantly higher in the high-arousal groups. For MS7, the Duration was found significantly lower in the high-arousal groups, and the MspatCorr was significantly higher in the high-arousal groups. Furthermore, all 9 microstates showed increased GFP in the high arousal groups as compared to the low groups. For the transition probability parameter, there were significant differences from MS1 to MS9, from MS3 to MS4 and MS5, and from MS6 to MS3.

For the valence dimension, there were fewer microstate parameters with significant differences. Only the MspatCorr of MS1 was observed to decrease significantly in the high valence groups compared with the low groups. As with the results of the arousal dimension, all 9 microstates showed increased GFP in the high valence groups as compared to the low groups. For the transition probability parameter, there were significant differences from MS2 to MS3, from MS4 to MS2, from MS6 to MS2, from MS7 to MS3, and from MS9 to MS1.

Furthermore, to be consistent with previous literatures (Shen et al., 2020; Hu et al., 2022a,b) for comparison, we repeated the microstate analysis on DEAP and SEED datasets and designated the number of microstates as 4 *a priori*. We also repeated the same statistical analysis on the DEAP dataset when the number of microstates was 4, as described in Section 2.3 (Statistical Analysis of Microstate Features). The results are shown in Supplementary Table S2 and Supplementary Figure S6. For the arousal dimension, the activities of microstate B and C were significantly increased in the high arousal groups compared to low arousal groups, while the activity of microstate D was significantly decreased in the high groups. Specifically, the Occurrence of B and C were significantly higher in the high-arousal groups. For microstate D, the Duration and Coverage was found significantly lower in the high-arousal groups. For the valence dimension, the activities of microstate B were significantly increased in the high valence groups, while the activity of microstate D was significantly decreased in the high groups. Specifically, microstate B showed increased Coverage in the high groups as compared to the low groups, while microstate D had decreased Duration.

### 3.3 Performance of emotion recognition

#### 3.3.1 Performance on SEED dataset

For the SEED dataset, EEG of each session contains 15 trials, each corresponding to a pre-given emotion label (5 trials for each category: negative, positive and neutral). The duration of each trial is about 4 min (the shortest is 178 s). We used the non-overlapping sliding windows to segment sub-epochs from each EEG recording. The optimal window length was determined to be 15 s according to the pre-experiment. To keep the number of sub-epochs in each trial consistent, we segmented sub-epochs from the first 178 s of each trial, so that 11 sub-epochs can be extracted from each trial, and 165 sub-epochs for each session.

**Figure 5:**
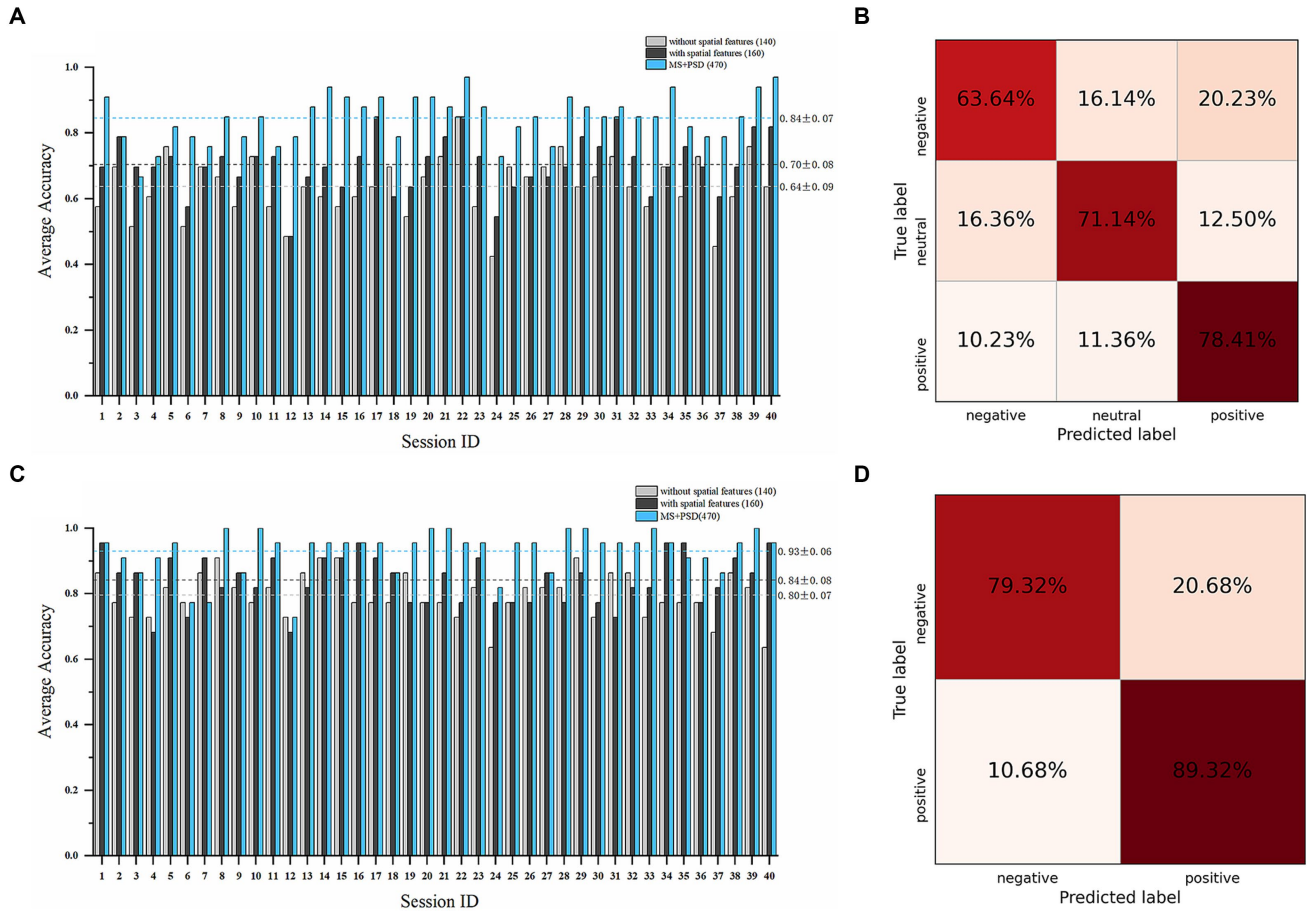
Emotion recognition performance on the SEED dataset. Average accuracy and confusion matrix (with all 160 microstate parameters) of the three-class classification experiment (positive vs. negative vs. neutral) are shown in **(A,B)**. Average accuracy and confusion matrix (with all 160 microstate parameters) of the binary classification experiment (positive vs. negative) are shown in **(C,D)** (Light grey bars: results with only 140 microstate temporal parameters; Dark grey bars: results with all 160 microstate temporal and spatial parameters; Blue bars: results with all 160 microstate parameters and 310 PSD features).

A total of 160 microstate features, including 140 temporal features and 20 spatial features, and 310 PSD features were obtained. We conducted subject-dependent experiments for three-class classification (positive vs. negative vs. neutral) and binary classification (positive vs. negative). The best-performing models in both experiments were WeightedEnsemble_L2, a weighted ensemble meta-model that implements ensemble selection and 2-layer stacking strategies. In the three-class classification (Figures 5A,B), when only 140 temporal features were used as features, the average accuracy of all sessions was 63.71% ± 8.85%; while when all the 160 temporal and spatial features were used, the average accuracy was 70.38% ± 8.03%, and the highest average accuracy was 81.82% on subject 8 and subject 15. When PSD features were incorporated with microstate features, the average accuracy increased to 84.47% ± 7.02%. In the binary classification (Figures 5C,D), the average accuracy of all sessions was 79.55% ± 6.82% with only 140 temporal features; while the average accuracy was 84.09 ± 7.54% with all the 160 microstate features, and the highest average accuracy was 93.18% on subject 6. When PSD features were incorporated, the average accuracy increased to 92.95% ± 6.66%.

#### 3.3.2 Performance on DEAP dataset

For the DEAP dataset, EEG of each subject contains 40 trials, and each trial is labeled in the dimension of arousal and valence. The duration of each trial is 60 s. Similarly, the non-overlapping sliding window method was used to segment sub-epochs from each trial. In this way, 4 sub-epochs can be extracted from each trial (some trials may not be long enough to extract 4 sub-epochs due to the removal of the bad segment in the preprocessing process). As a result, 160 sub-epochs can be extracted from EEG of each subject.

**Figure 6:**
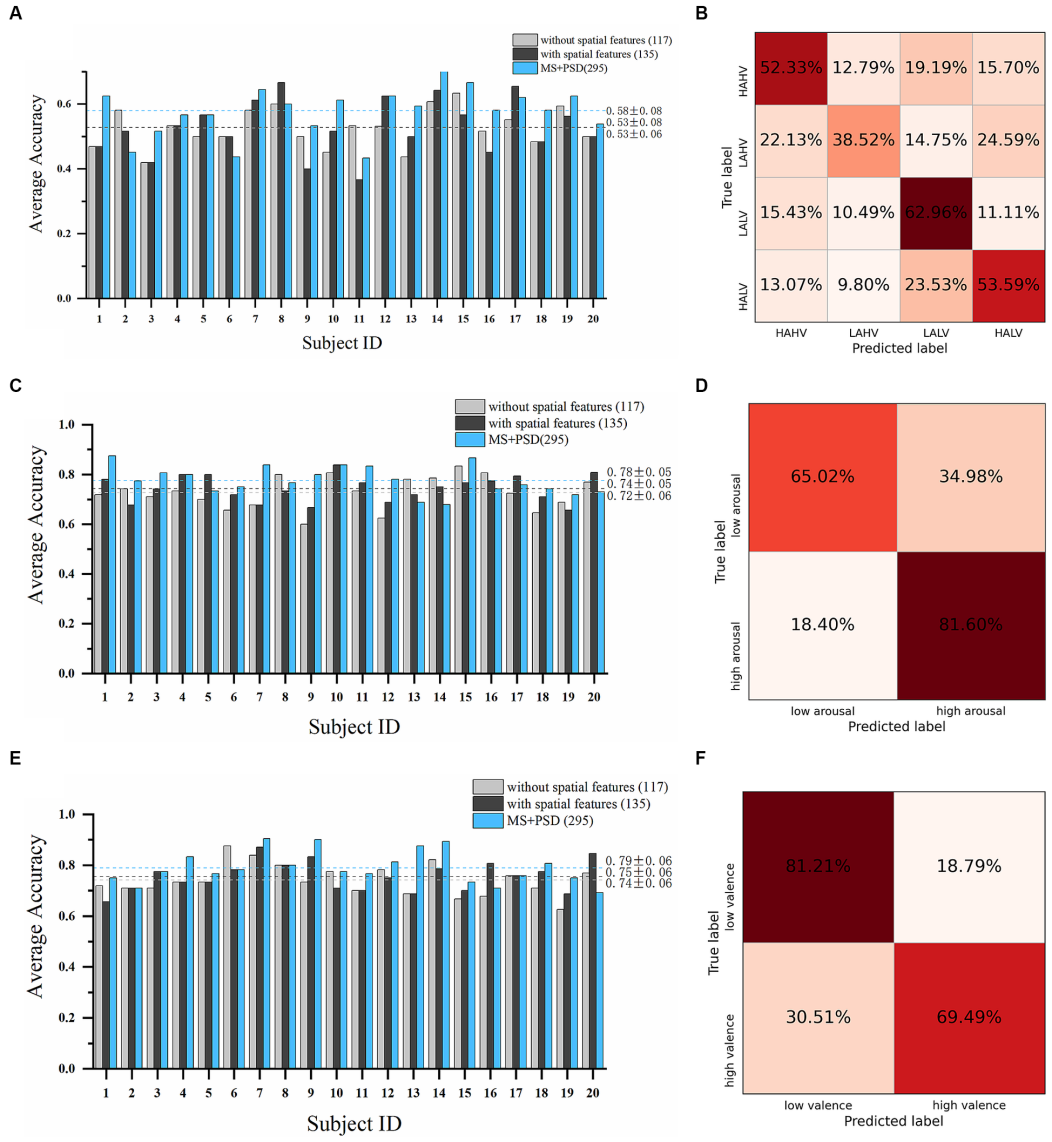
Emotion recognition performance on the DEAP dataset. Average accuracy and confusion matrix (with all 135 microstate parameters) of the four-class classification experiment are shown in **(A,B)**. Results in arousal dimension are shown in **(C,D)**. Results in valence dimension are shown in **(E,F)** (Light grey bars: results with only 117 microstate temporal parameters; Dark grey bars: results with all 135 microstate temporal and spatial parameters; Blue bars: results with all 135 microstate parameters and 160 PSD features).

A total of 135 microstate features, including 117 temporal features and 18 spatial features, and 160 PSD features were obtained. We conducted subject-dependent experiments for four-class classification (HAHV vs. HALV vs. LAHV vs. LALV) and binary classification (arousal dimension and valence dimension). The best-performing model in these experiments were also WeightedEnsemble_L2. In the four-class classification experiment (Figures 6A,B), when only the 117 temporal features were used, the average accuracy of all subjects was 52.61% ± 5.81%; while when all the 135 temporal and spatial features were used, the average accuracy was 52.77% ± 8.29%, and the highest average accuracy was 66.67% on subject 8. When PSD features were incorporated with microstate features, the average accuracy increased to 58.02% ± 8.07%. In the arousal dimension (Figures 6C,D), the average accuracy of all subjects was 72.67% ± 6.37% with only 117 temporal features; while the average accuracy was 74.33% ± 5.17% with all the 135 microstate features, and the highest average accuracy was 83.87% on subject 10. When PSD features were incorporated, the average accuracy increased to 77.61% ± 5.44%. In the valence dimension (Figures 6E,F), the average accuracy of all subjects was 74.11% ± 6.02% with only 117 temporal features; while the average accuracy was 75.49% ± 5.70% with all the 135 microstate features, and the highest average accuracy was 87.10% on subject 7. When PSD features were incorporated, the average accuracy increased to 78.95% ± 6.20%.

The performance in this paper was also compared with previous studies on subject-dependent emotion recognition using SEED (Table 2) and DEAP (Table 3) datasets. The results showed that, compared with other recent studies that also used these datasets for subject-dependent emotion recognition, we achieved higher accuracy.

**Table 2:** The subject-dependent emotion recognition accuracies of different feature sets on the SEED dataset (Standard deviation shown in parentheses.)

Studies	Feature set	Classifier	Accuracy	
			Three classes (%)	Binary classes (%)
Wang et al. (2019)	Differential entropy	P-GCNN	84.08 (8.50)	N/A
Wang et al. (2019)	Rational asymmetry	P-GCNN	84.35 (10.28)	N/A
Val-Calvo et al. (2019)	Oscillatory features	Machine learning models*	82.3 (4.4)	N/A
Kim et al. (2022)	Wavelet packet transform	LSTM	81.07 (5.36)	N/A
Du et al. (2022)	Differential entropy	ATDD-LSTM	79.26 (12.79)	N/A
**This study**	Microstate temporal and spatial parameters	AutoGluon	70.38 (8.03)	84.09 (7.54)
	**Microstate parameters and power spectral density**	**AutoGluon**	**84.47 (7.02)**	**92.95 (6.66)**

P-GCNN, PLV-based graph convolutional neural network; LSTM, long short-term memory; ATDD-LSTM, attention-based LSTM with domain discriminator; *The article used 8 kinds of machine learning models for classification, but the best-performing model was not reported. N/A, not available. Bold values indicate the best-performing results across studies.

**Table 3:** The subject-dependent emotion recognition accuracies of different feature sets on the DEAP dataset (Standard deviation shown in parentheses).

Studies	Feature set	Classifier	Accuracy		
			Four classes (%)	Arousal (%)	Valence (%)
Zhuang et al. (2017)	Intrinsic mode functions (IMFs)	SVM	N/A	72.10 (7.15)	70.41 (7.05)
Wang et al. (2019)	Differential entropy	P-GCNN	N/A	77.03 (11.49)	73.31 (11.66)
Lew et al. (2020)	Temporal dependencies and spatial dependencies	RODAN	38.16 (10.0)	63.97 (11.40)	62.93 (8.20)
Li et al. (2021)	Power spectral density	AdaBoost fusion	N/A	59.00 (10.74)	70.25 (8.25)
Li et al. (2023)	Spatial-spectral features (Welch method)	TARDGCN	N/A	58.12	57.73
Li et al. (2024)	Preprocessed EEG	Fractal-SNN	N/A	69.61	69.84
**This study**	Microstate temporal and spatial parameters	AutoGluon	52.77 (8.29)	74.33 (5.17)	75.49 (5.70)
	**Microstate parameters and power spectral density**	**AutoGluon**	**58.02 (8.07)**	**77.61 (5.44)**	**78.95 (6.20)**

SVM, support vector machine; P-GCNN, PLV-based graph convolutional neural network; RODAN, regionally-operated domain adversarial network; TARDGCN, trainable adjacency relation driven graph convolutional network; Fractal-SNN, fractal spike neural network; N/A, not available. Bold values indicate the best-performing results across studies.

## 4 Discussion

In this study, we used microstate analysis to investigate the temporal and spatial dynamics of emotional EEG signals, and further tested the feasibility and effectiveness of microstate approach on classification of emotions. The key challenge of applying the microstate method to emotion is the determination of the optimal number of microstates adaptively. We proposed a KL_GEV_ criterion to automatically and adaptively identify this optimal number in emotional EEG signals. In our study, the proposed KL_GEV_ criterion revealed 10 microstates best described the SEED dataset (Figure 3B), and 9 microstates for the DEAP dataset (Figure 3D). The results indicate that EEG data in emotional states may need more microstates to describe compared to resting state, which contains the dynamics of emotion and other emotion-related cognitive processes.

### 4.1 EEG microstates: the basic building blocks of emotion cognition

In resting-state EEG signals, researchers commonly identify four canonical microstates, which were consistently labeled by Koenig et al. (1999) as class A, B, C, and D. According to the topography similarity, we related some microstates in our study to four canonical microstates and the new labeling system in Tarailis et al. (2021, 2023), and found an excellent correspondence. MS4, MS9 in SEED dataset and MS4, MS6 in DEAP dataset are similar to map A, which exhibit left posterior–right anterior orientation. MS3 in both datasets are similar to map B, which exhibit right posterior–left anterior orientation. MS2 in SEED and MS1 in DEAP are similar to map C, which exhibit anterior–posterior orientation. MS1, MS10 in SEED and MS8 in DEAP are similar to map D, which exhibit fronto-central maximum. MS6 in SEED and MS2, MS9 in DEAP can be viewed as map E, which exhibit local maxima in posterior. MS7 in both datasets can be viewed as map F, and MS8 in SEED and MS5 in DEAP dataset can be viewed as map G.

Besides, there is a clear microstates correspondence between SEED and DEAP datasets (Figure 4). This one-to-one correspondence indicates that there are some consistent functional microstates in emotion-related EEG signals despite different experimental conditions. These identical functional microstates may reflect the functional patterns of the brain in the process of emotional cognition, and correspond to the basic building blocks in emotion-related information processing. Tarailis et al. (2023) provides a comprehensive review on the functional aspects of EEG microstates. According to this review, microstate A is associated with the auditory-language network and links to subjects’ arousal/arousability, and the spatially correlated brain regions include bilateral superior and middle temporal gyri (Custo et al., 2017). Microstate B shows associations with the visual network and is also related to self-visualization, autobiographical memory, and scene visualization. It is thought to be spatially correlated with bilateral occipital areas (Custo et al., 2017). Both the SEED and DEAP datasets use auditory and visual stimuli, as a result, microstate A (MS4, MS9 in SEED and MS4, MS6 in DEAP) and microstate B (MS3 in both datasets) may play foundational and stimuli-related roles in emotional cognition. Microstate C is related to processing personally significant information, self-reflection, and self-referential internal mentation. Microstate E (frequently merged with microstate C) plays important roles in processing interoceptive and emotional information, and is associated with the salience network. Both microstates C (MS2 in SEED and MS1 in DEAP) and E (MS6 in SEED and MS2, MS9 in DEAP) are spatially correlated with the cingulate cortex and limbic system, which are known to be essential brain areas in emotional information processing (Phan et al., 2002; Rolls, 2019). This accounts for the significantly dominant Coverage and Occurrence of microstates C and E in both datasets, regardless of particular emotional conditions. Microstate D is associated with executive functioning, including working memory and attention. It is thought to be spatially correlated with the dorsal attention network, including right-lateralized frontal and parietal areas (Custo et al., 2017). Studies (Thiruchselvam et al., 2012; Storbeck and Watson, 2014) have suggested that emotion and working memory domains are integrated, such that positive affect enhances verbal working memory, whereas negative affect enhances spatial working memory. These high-level cognitive functions may have reciprocal connectedness allowing for bidirectional influence. The relatively dominant Coverage and Occurrence of microstates D (MS1, MS10 in SEED and MS8 in DEAP) demonstrated these findings. Little is known about microstate F and G. Microstate F is suggested to be a part of the default mode network, which was found to consistently decrease its activity in task states (Raichle, 2015). This may account for the relatively few Coverage and Occurrence of microstate F (MS7 in both datasets) in all emotional conditions. Microstate G is potentially linked to the somatosensory network, which was found involved in the cognition of some basic emotions (Tettamanti et al., 2012).

The functional interpretability of EEG microstate enables us to have a deeper understanding of spatiotemporal dynamics of whole-brain activity during emotional cognition, which is a unique advantage compared to other EEG features. These findings further prove the effectiveness of the proposed KL_GEV_-criterion-based method, which can identify several consistent emotion-related microstates from different emotional EEG datasets. However, the relationship between specific microstates and specific cognitive and affective processes still needs further study to provide a more comprehensive insights of emotion cognition.

### 4.2 Modulation of microstates by emotion

The statistic results (Supplementary Figures S3–S5) indicate that these temporal and spatial parameters reveal the characteristics of brain activity under different emotional states with excellent temporal resolution (within milliseconds), while retaining certain spatial information of the EEG signal. In addition, we find that the microstate parameters with significant differences in different emotional states have apparent patterns, and these patterns are helpful to reveal the specific relationship between microstates and emotion. These differences reflect the temporal and spatial dynamics of whole-brain activity during different affective processes, and reveal the changes in the functional states of underlying neural assemblies in the brain while listening to emotional music or watching emotional movie clips. Furthermore, these results provide a novel feature set and theoretical support for the subsequent emotion recognition.

**Figure 7:**
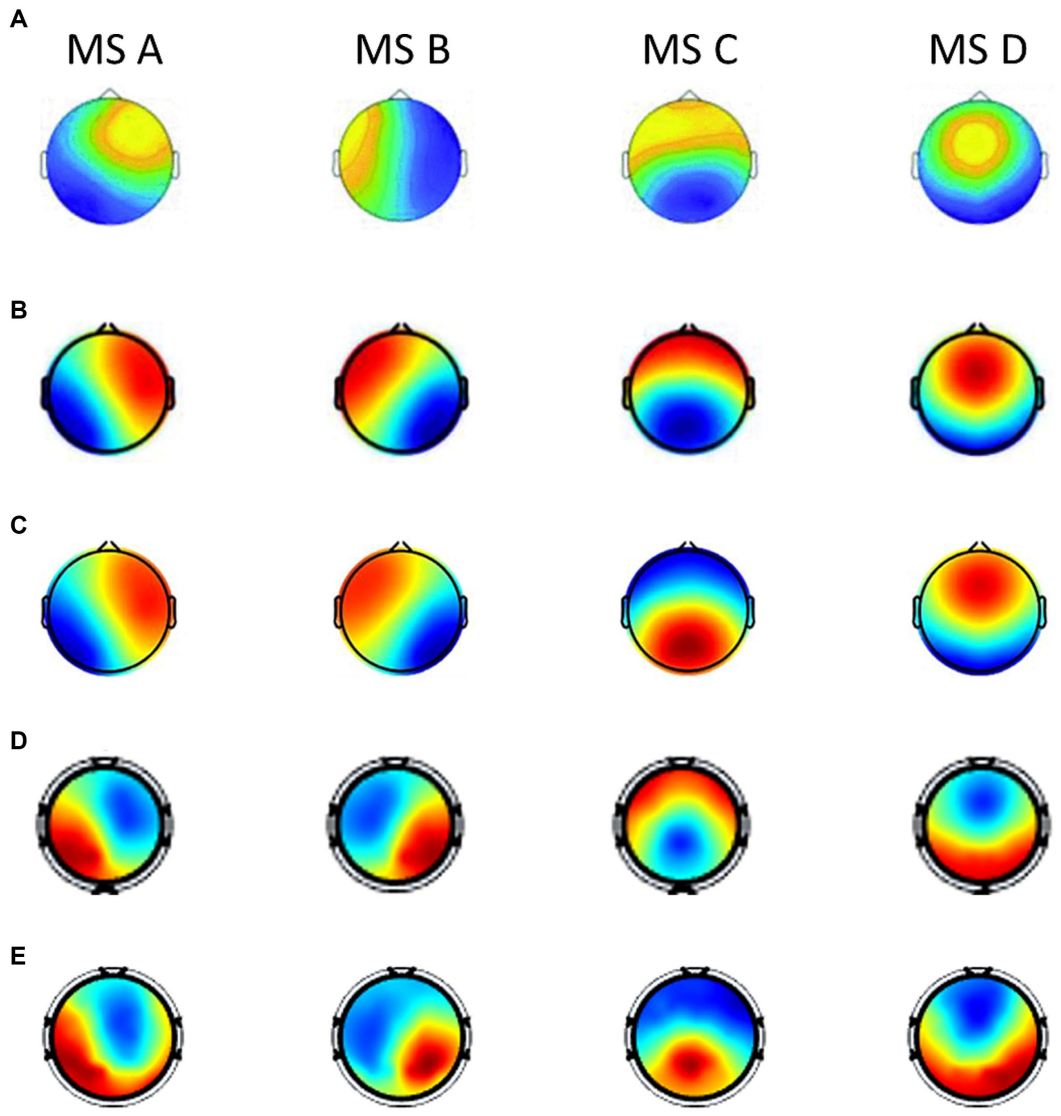
The topographical maps of the microstates across different studies when the optimal number of microstates is defined as 4 (labeled as MS A, MS B, MS C, and MS D). **(A)** Microstate maps in Shen et al. (2020). There were results of different frequency bands in the article, we choose the results of broad-band (1–30 Hz) for consistency of comparison. **(B)** Microstate maps in Hu et al. (2022a). There were results of different topographical clustering strategies in the article, we choose the results of the recommended strategy (case 3). **(C)** Microstate maps in Hu et al. (2022b). **(D)** Microstate maps of the DEAP dataset in our study. **(E)** Microstate maps of the SEED dataset in our study.

In order to further analyze the changing rules of microstates and parameters under different emotional states, and have a better understanding of the neurophysiological significance of microstates during the cognitive process of emotion, we summarize the current studies that also employ the microstate analysis method to emotional EEG signals (Shen et al., 2020; Hu et al., 2022a,b). These three studies conducted the microstate and statistical analysis on the DEAP dataset with one accord. The topographical maps of the microstates across these studies were shown in Figure 7. It can be seen that the four microstates obtained in DEAP and SEED datasets in this paper resemble the four canonical microstate topographic maps in the previous studies (Khanna et al., 2015; Michel and Koenig, 2018), and share a strong similarity with the other three studies. Different microstates and their underlying brain sources play different roles in different emotional cognition processes. Several recent findings show that the four canonical microstates have strong electrophysiological correspondence with four important functional brain networks observed from functional magnetic resonance imaging (fMRI) and EEG source localization (Custo et al., 2017): auditory network, visual network, anterior regions of default mode network (DMN) and dorsal attention network (DAN).

We summarize the statistic results of these three studies, and compare with our study, which is shown in Table 4. As summarized in Table 4A, for the arousal dimension, we drew the consistent conclusions with (Hu et al., 2022a,b): the activity of microstate C increased significantly in the high arousal groups, while the activity of microstate D increased significantly in the low arousal groups, that is, the arousal ratings of subjects were positively correlated with the activity of microstate C (anterior regions of the default mode network) and negatively correlated with microstate D (dorsal attention network). For the valence dimension (as summarized in Table 4B), we had the consistent conclusions with (Shen et al., 2020; Hu et al., 2022a,b): the activity of microstate B increased significantly in the high valence groups, while the activity of microstate D increased significantly in the low valence groups, in other words, the valence ratings of subjects were positively correlated with the activity of microstate B (visual network) and negatively correlated with microstate D. These findings are congruent with the observations of how emotion arousal and valence modulate the activities of functional brain networks in the previous studies (Mourao-Miranda et al., 2003; Posner et al., 2009; Colibazzi et al., 2010). These results partially revealed the specific neurophysiological significance of microstates during the emotional cognitive process, and broaden our knowledge of the functional interpretability of EEG microstates.

**Table 4:** Summary of published studies regarding how EEG microstates are modulated by different emotion states using the public DEAP dataset and restricting the analysis to the four canonical microstate maps.

Study	Dataset	Microstate			
		A	B	C	D
(A) Comparison on the arousal dimension					
Shen et al. (2020)	DEAP (1–30 Hz)				
Hu et al. (2022a)	DEAP (1–45 Hz)			↑ Dur	↓ Cov, Occ
Hu et al. (2022b)	DEAP (1–45 Hz)			↑ Cov, Occ	
This study	DEAP (1–45 Hz)		↑ Occ	↑ Occ	↓ Cov, Dur
(B) Comparison on the valence dimension					
Shen et al. (2020)	DEAP (1–30 Hz)	↓ Occ	↑ Cov, Dur		↓ Cov, Occ
Hu et al. (2022a)	DEAP (1–45 Hz)	↑ Dur	↑ Dur		↓ Cov, Occ
Hu et al. (2022b)	DEAP (1–45 Hz)				↓ Occ
This study	DEAP (1–45 Hz)		↑ Cov		↓ Dur

Since the SEED dataset is not based on the dimensional emotion model, the results for the SEED dataset are not included in this table. Comparison on the (A) arousal and (B) valence dimension. Dur, Duration; Occ, Occurrence; Cov, Coverage. The arrows indicate the significant changes in high arousal (or valence) groups compared to low groups.

### 4.3 Limitations and future directions

However, the neurophysiological significance of microstates summarized in this paper is based on conclusions of the four canonical microstates and resting-state brain networks, so there is still a lack of studies locating emotion-related EEG microstates to specific functional brain networks, which will limit the interpretability of EEG microstate in emotion cognitive process. A more comprehensive and accurate correlation between EEG microstate at emotional states and brain functional networks remains an open issue. For example, the combination of fMRI and EEG source localization may help researchers to have a deeper understanding of EEG microstates and emotion.

As can be seen from Figures 5, 6, Tables 2, 3, the introduced microstate spatial features further improve the accuracy of emotion recognition on the basis of temporal features, which compensate for the spatial information of EEG signals. In addition, with a larger number of features and additional frequency domain information, the performance is significantly enhanced with both PSD and microstate features. In future studies, it may be a great idea to use both microstate features and frequency domain features for emotion recognition, which are complementary to each other. These results indicate that the feature set of microstate parameters in this paper can effectively capture the emotion-related characteristics of EEG signals, thus improving the accuracy of emotion recognition. However, preprocessing procedure (e.g., judgment of bad channels, judgment of artifacts such as eye movement and muscle artifacts during ICA), experimental settings (e.g., how labels are assigned, and how datasets are partitioned), feature extraction methods and dimensions of features (and whether feature selection is performed), and classifiers (machine learning models or deep learning models), etc., vary a lot across different studies. These factors have a great impact on the final classification performance. Such cross-study comparisons may not be so straightforward and fair.

What’s more, compared to the state-of-the-art studies using deep learning and other features for emotion recognition, our results of employing microstate parameters and machine learning models are less competitive. We must acknowledge the powerful feature extraction and classification capabilities of deep learning, which significantly improve the performance of EEG emotion recognition. As a future direction, we are trying to develop a deep or broad artificial neural network, which is special for the microstate sequences, to improve the performance of EEG-based classification tasks not limited to emotion recognition. In this way, we may benefit from both the functional interpretability of microstate features and the powerful classification capabilities of deep learning.

## 5 Conclusion

The main purpose of this study is to investigate the temporal and spatial dynamics of emotional EEG signals and the specific neurophysiological significance of microstates during the emotion cognitive process, and further explore the feasibility and effectiveness of applying novel features based on EEG microstates to emotion recognition. Determining the optimal number of microstates automatically is the key challenge of applying the microstate analysis method to emotion. To address the challenge, we proposed a KL_GEV_ criterion, which can automatically and adaptively identify the optimal number of microstates in emotional EEG signals. Also, we found the relationship between microstates and specific emotions, which broaden our knowledge of the interpretability of emotional EEG microstates. In summary, the findings in this work demonstrate the effectiveness of the proposed KL_GEV_-criterion-based method in researching emotional EEG signals, and the microstate features are novel and promising feature sets for EEG-based emotion recognition. We hope this work will stimulate future research in: (1) further investigating the specialized roles of EEG microstates in explaining the dynamics of emotion, e.g., the combination of fMRI and EEG source localization may help deeper understanding of EEG microstates and emotion, (2) developing novel deep neural networks based on microstate sequences, to improve the performance of EEG-based emotion recognition.

## Data availability statement

The datasets presented in this study can be found in online repositories. The names of the repository/repositories and accession number(s) can be found below: SEED Dataset (https://bcmi.sjtu.edu.cn/~seed/index.html) and DEAP: a Dataset for Emotion Analysis using Physiological and Audiovisual Signals (http://www.eecs.qmul.ac.uk/mmv/datasets/deap/index.html).

## Ethics statement

Ethical approval was not required for the studies involving humans because we use publicly available datasets in this study. The studies were conducted in accordance with the local legislation and institutional requirements. The participants provided their written informed consent to participate in this study.

## Author contributions

ZW: Conceptualization, Data curation, Methodology, Software, Writing – original draft, Writing – review & editing. HoL: Conceptualization, Data curation, Supervision, Writing – review & editing, Funding acquisition, Methodology. LM: Conceptualization, Funding acquisition, Methodology, Supervision, Writing – review & editing, Writing – original draft. HaL: Conceptualization, Funding acquisition, Methodology, Supervision, Writing – review & editing.
