# Supplementary material for: Emotion recognition based on microstate analysis from temporal and spatial patterns of electroencephalogram
Source: Front Neurosci. 2024 Mar 14;18:1355512. doi: 10.3389/fnins.2024.1355512 (PMC10972890; doi:10.3389/fnins.2024.1355512)
Supplement: Supplementary file 1 [file Data_Sheet_1.PDF]

Supplementary Material

Emotion Recognition Based on Microstate Analysis from Temporal and Spatial Patterns of Electroencephalogram

Zhen Wei, Hongwei Li, Lin Ma, Haifeng Li*

*** Correspondence:** Haifeng Li: lihaifeng@hit.edu.cn

1 Supplementary Figures and Tables

1.1 Supplementary Figures

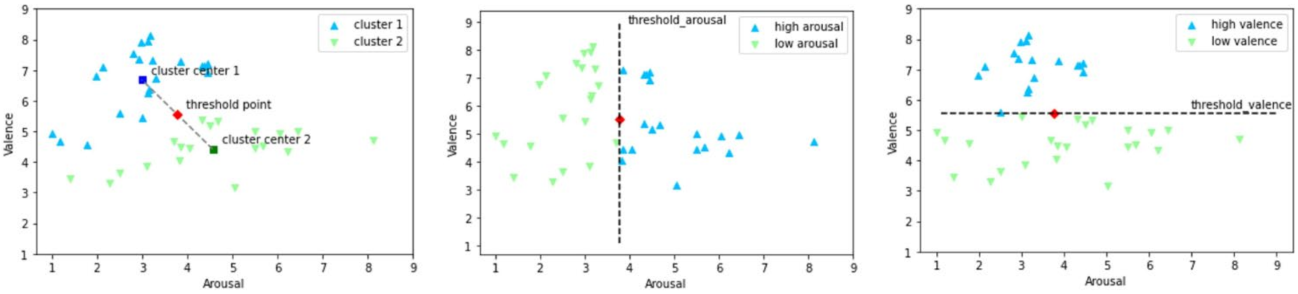

A B C

Supplementary Figure 1. An example of the self-adaptive threshold reassignment method and determination of adaptive thresholds and target emotion classes for subject 3 in DEAP: **(A)** Results of k-means clustering, the blue square and green square are the cluster centers, and the red diamond is the middle point (threshold point). **(B)** Target classes for arousal dimension. **(C)** Target classes for valence dimension.

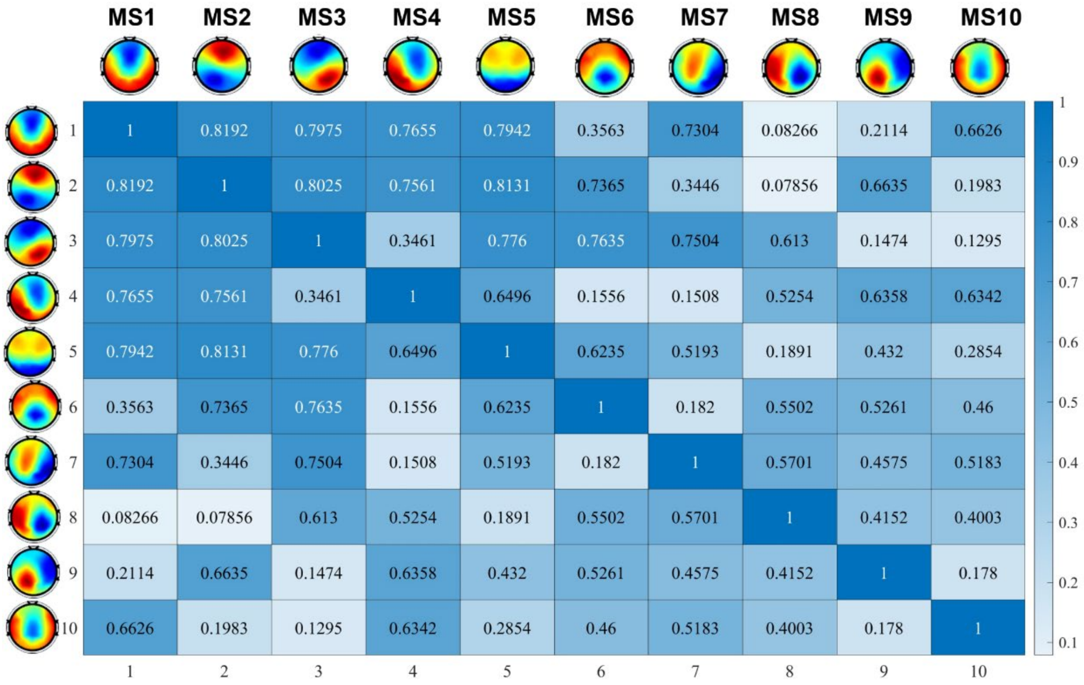

(A)

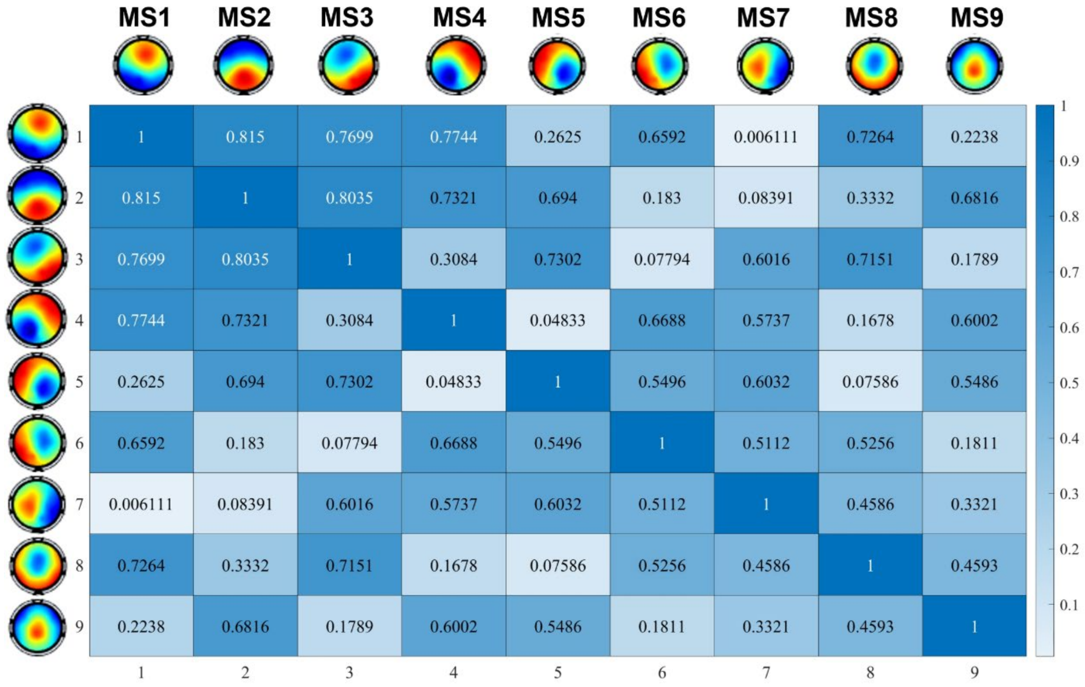

(B)

Supplementary Figure 2. The Pearson correlation coefficients between microstates in **(A)** SEED dataset and **(B)** DEAP dataset.

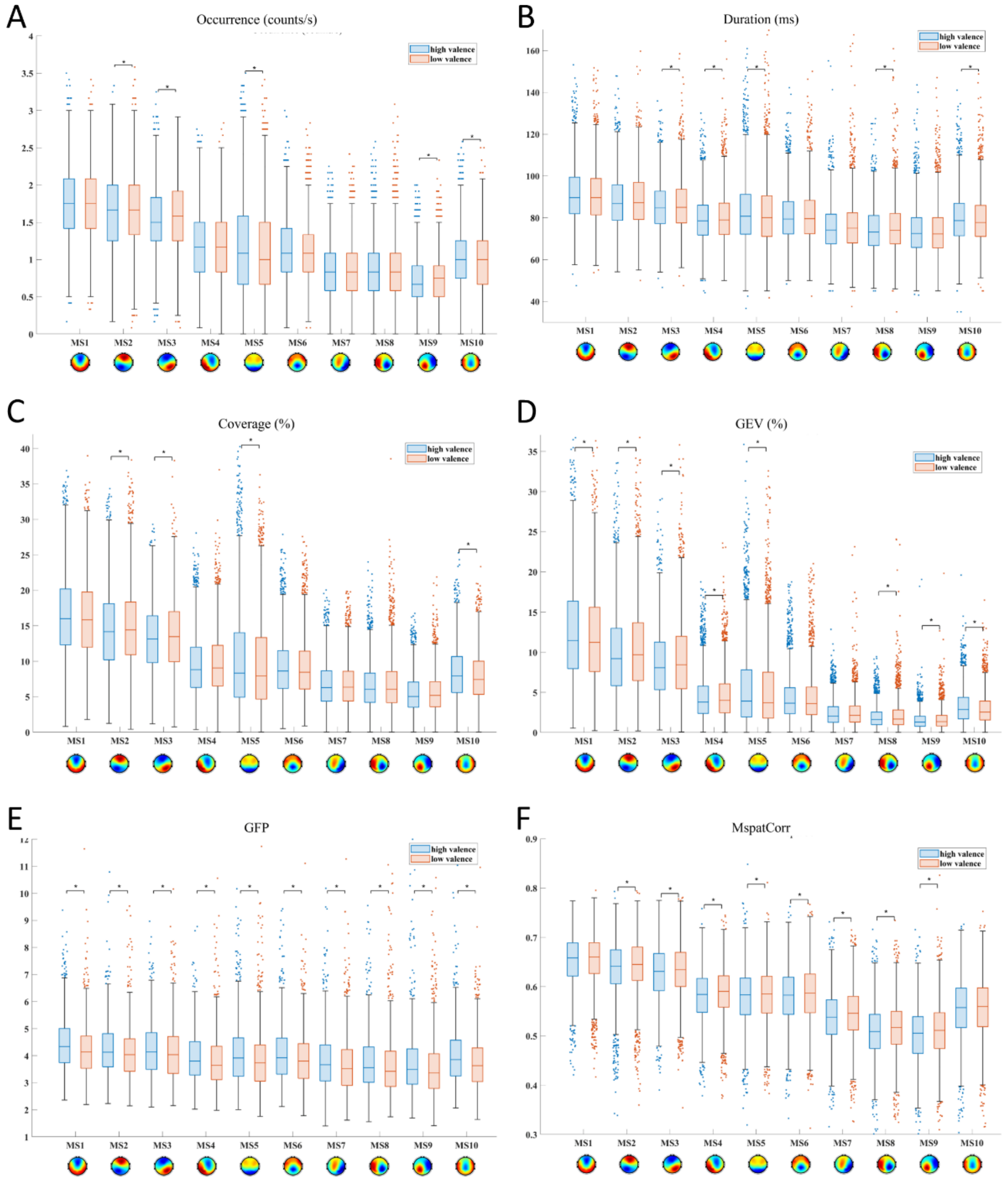

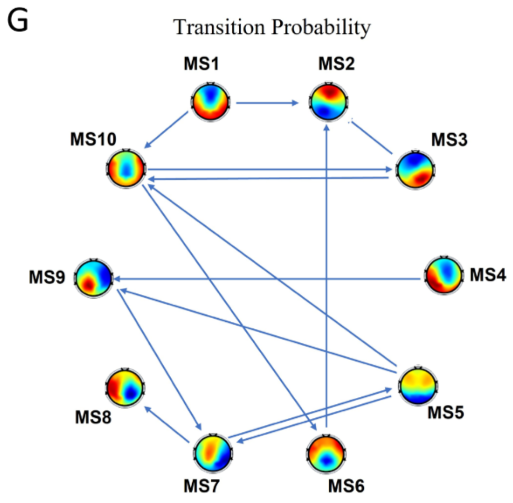

Supplementary Figure 3. Wilcoxon rank-sum test of microstate features for SEED dataset to examine the statistical differences in EEG microstate activities between positive and negative groups. In the statistical results of **(A)** Occurrence (counts/s), **(B)** Duration (ms), **(C)** Coverage (%), **(D)** GEV (%), **(E)** GFP and **(F)** MspatCorr, the dots and the box plots represent the original feature distribution. In the statistical results of **(G)** Transition probability, the blue arrows indicate significant difference from one microstate to another. (* $P < 0.05$).

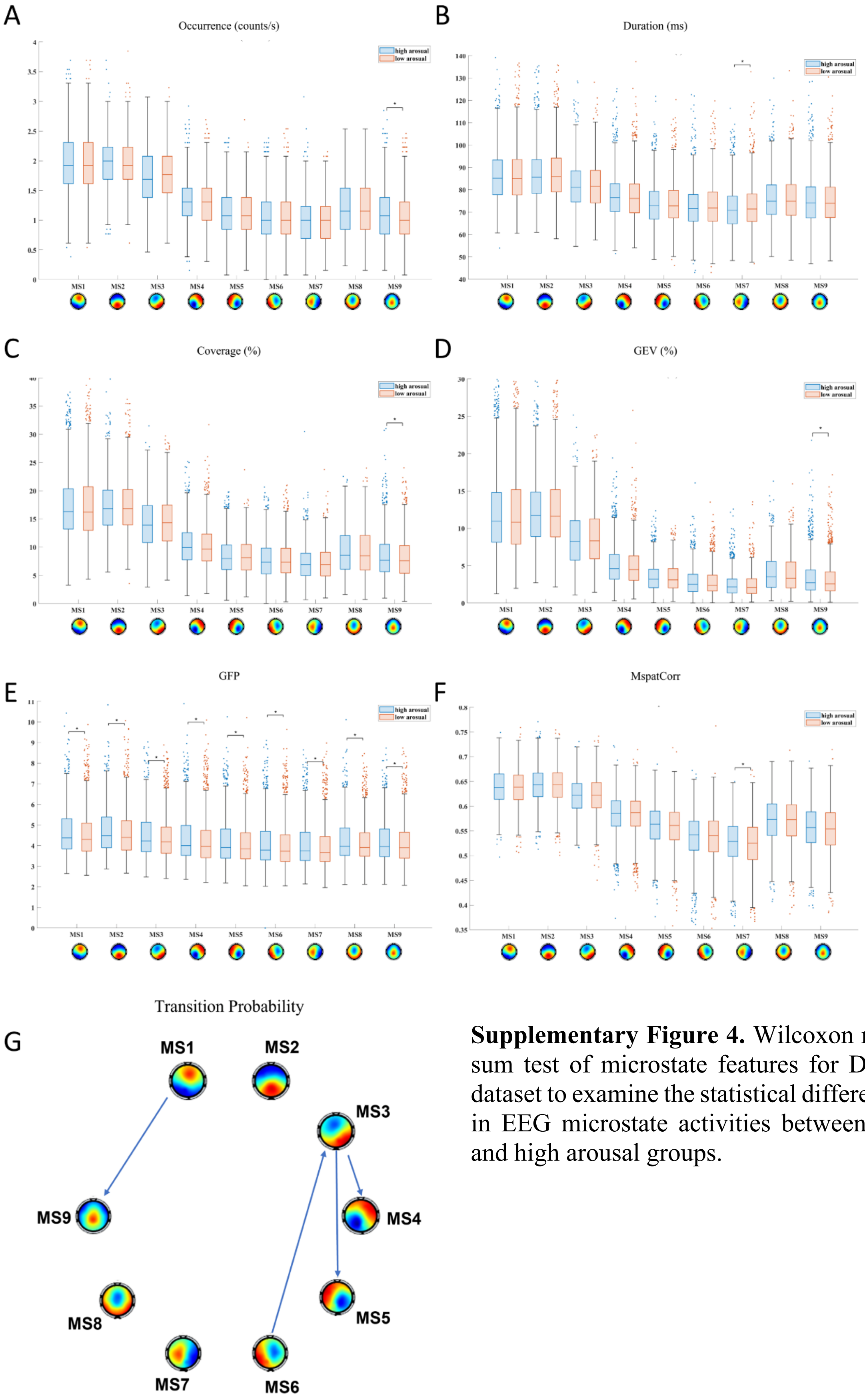

Supplementary Figure 4. Wilcoxon rank-sum test of microstate features for DEAP dataset to examine the statistical differences in EEG microstate activities between low and high arousal groups.

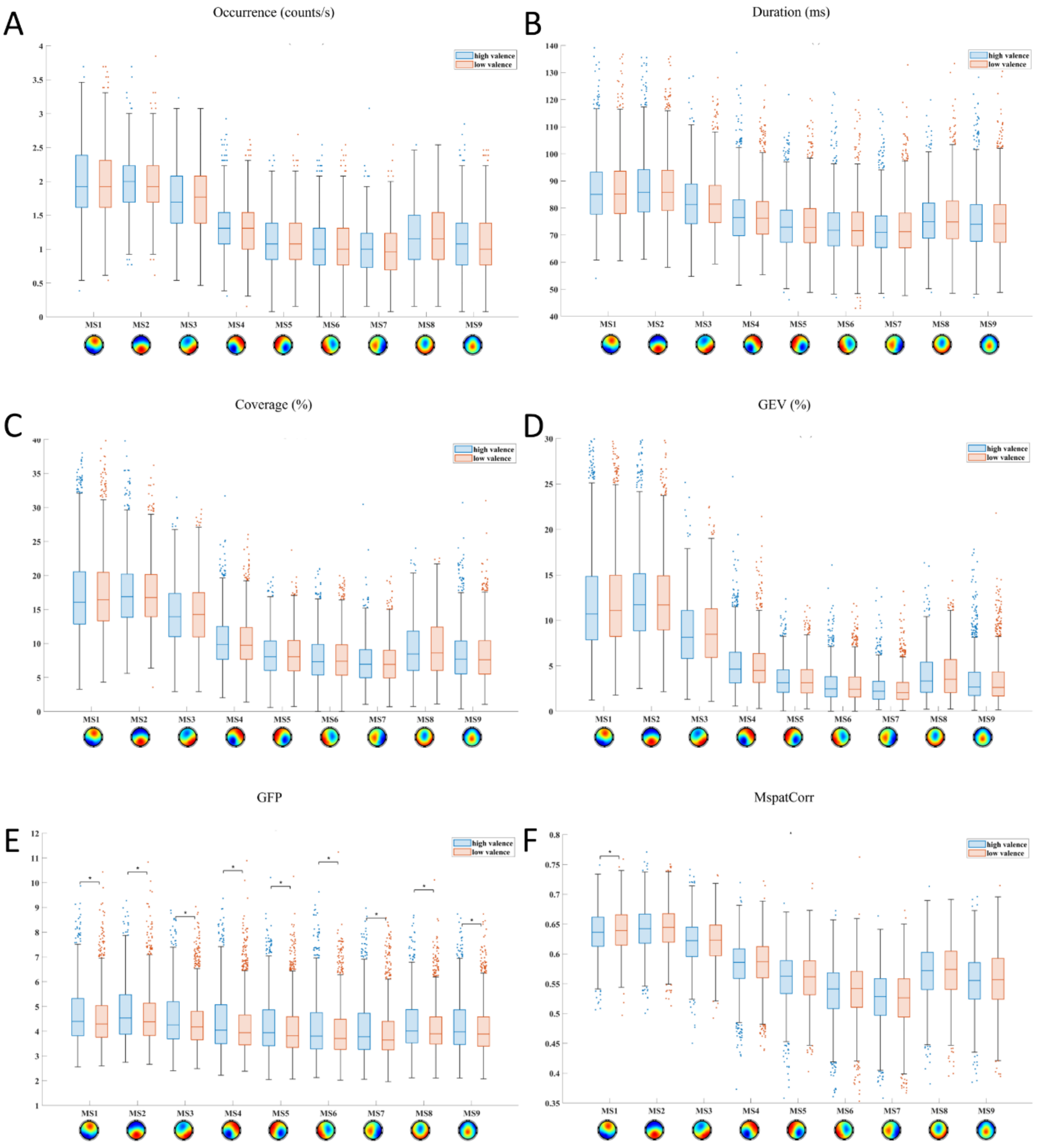

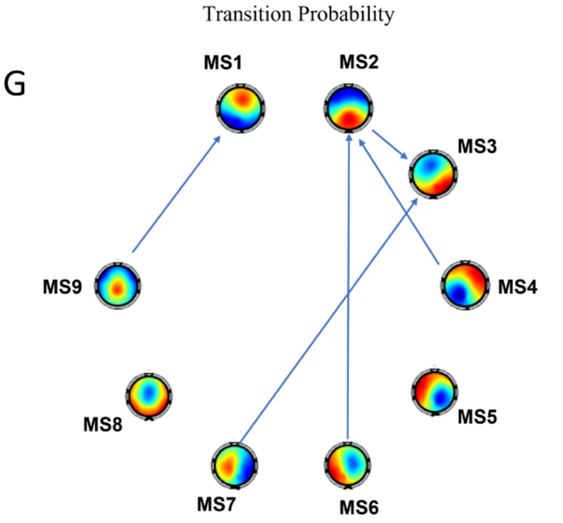

Supplementary Figure 5. Wilcoxon rank-sum test of microstate features for DEAP dataset to examine the statistical differences in EEG microstate activities between low and high valence groups.

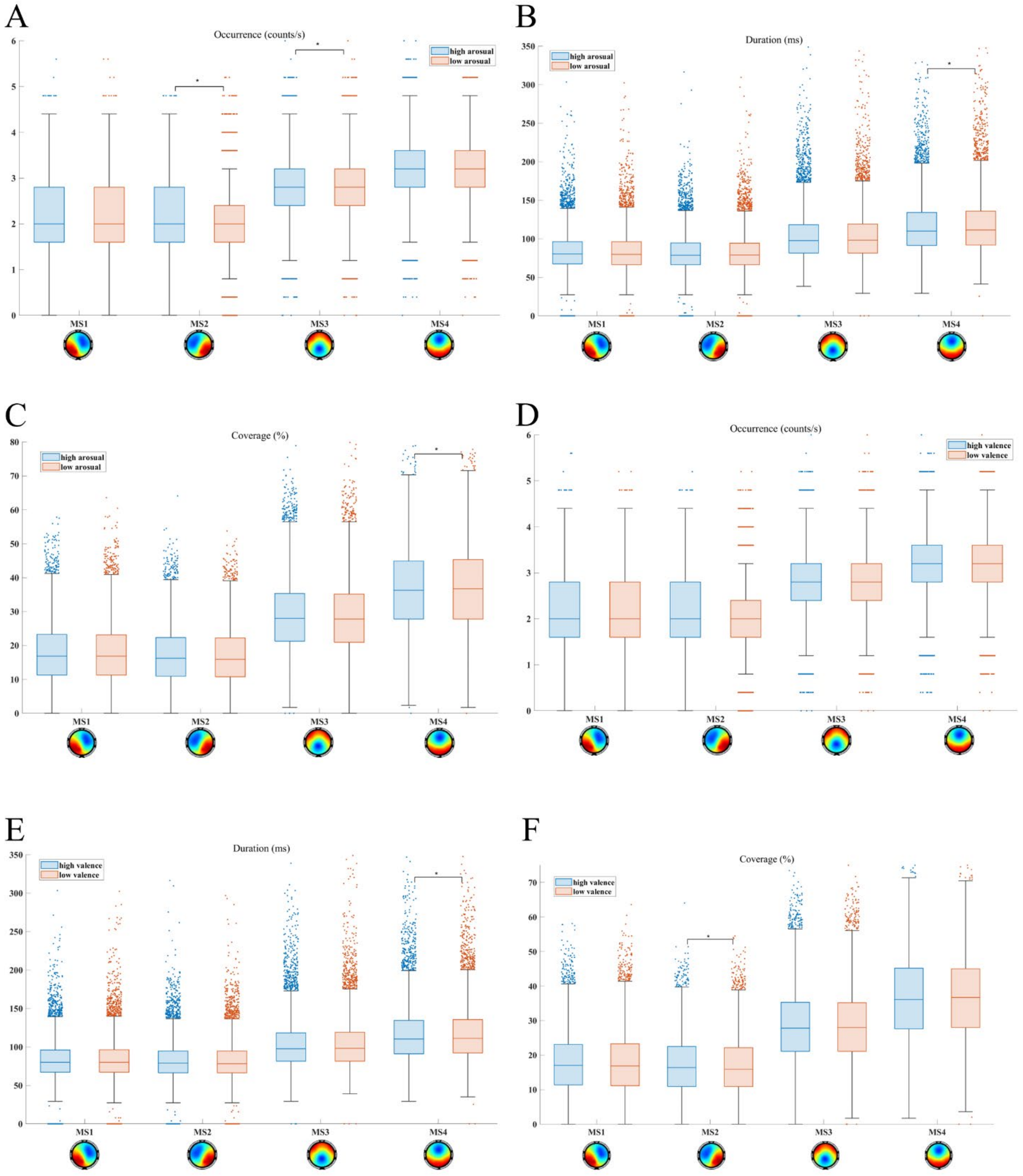

Supplementary Figure 6. The results of Wilcoxon rank-sum test for DEAP dataset when the number of microstates is designed as 4. In the statistical results of **(A)** Occurrence in arousal dimension, **(B)** Duration in arousal dimension, **(C)** Coverage in arousal dimension, **(D)** Occurrence in valence dimension, **(E)** Duration in valence dimension, and **(F)** Coverage in valence dimension, the dots and the box plots represent the original parameter distribution. (* $P \leqslant 0.05$)

1.2 Supplementary Tables

Supplementary Table 1. The self-adaptive ratings thresholds on arousal and valence dimensions for each subject in DEAP.

Subject ID	Arousal	Valence	Subject ID	Arousal	Valence
1	5.68	5.23	12	6.36	4.97
2	5.61	6.02	13	6.66	4.86
3	3.78	5.55	14	5.44	4.96
4	4.59	4.65	15	4.72	5.85
5	5.17	4.98	16	4.97	4.03
6	4.66	5.76	18	5.68	5.63
7	4.95	4.72	19	5.40	5.23
9	5.68	5.46	20	5.62	5.82
10	4.97	5.42	21	6.04	5.66
11	4.17	5.09	22	5.35	5.12
Trials of high arousal		427	Trials of high valence		386
Trials of low arousal		373	Trials of low valence		414
Mean threshold of arousal ratings					5.28
Mean threshold of valence ratings					5.25

Supplementary Table 2. The results of Wilcoxon rank-sum test for DEAP dataset when the number of microstates is designed as 4. The table shows the mean value (standard deviation) and p value for Occurrence, Duration and Coverage parameters of **(A)** arousal and **(B)** valence dimensions.

		**Microstate classes**			
**Parameters**		A 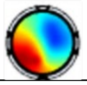	B 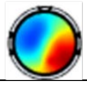	C 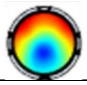	D 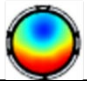
		**(A) Arousal**			
**Occurrence (counts/s)**	High	2.09 (±0.86)	2.05 (±0.83)	2.81 (±0.77)	3.16 (±0.77)
	Low	2.08 (±0.86)	2.03 (±0.84)	2.78 (±0.78)	3.16 (±0.78)
	P	0.48	**0.05***	**0.02***	0.68
**Duration (ms)**	High	83.78 (±26.07)	82.50 (±24.27)	103.62(±32.20)	116.13(±35.58)
	Low	83.84 (±27.22)	82.72 (±25.60)	103.98(±33.34)	117.81(±37.74)
	P	0.37	0.78	0.75	**0.02***
**Coverage (%)**	High	17.78 (±8.89)	17.04 (±8.83)	28.84 (±10.65)	36.34 (±12.12)
	Low	17.75 (±9.15)	16.89 (±8.25)	28.63 (±10.82)	36.73 (±12.46)
	P	0.37	0.29	0.16	**0.05***
		**(B) Valence**			
**Occurrence (counts/s)**	High	2.08 (±0.86)	2.05 (±0.84)	2.80 (±0.78)	3.16 (±0.77)
	Low	2.08 (±0.86)	2.03 (±0.83)	2.80 (±0.77)	3.16 (±0.77)
	P	0.76	0.12	0.79	0.91
**Duration (ms)**	High	83.74 (±26.43)	82.70 (±24.69)	103.60(±32.27)	116.48(±36.56)
	Low	83.87 (±26.79)	82.50 (±25.09)	103.97(±33.17)	117.32(±36.65)
	P	0.93	0.55	0.47	**0.05***
**Coverage (%)**	High	17.74 (±8.89)	17.08 (±8.31)	28.73 (±10.78)	36.45 (±12.41)
	Low	17.78 (±9.12)	16.88 (±8.27)	28.76 (±10.68)	36.59 (±12.16)
	P	0.83	**0.05***	0.74	0.27

The asterisk indicates significant difference ($p \leq 0.05$).
